# Supplementary figures and images for: SLC38A2 provides proline to fulfill unique synthetic demands arising during osteoblast differentiation and bone formation
Source: eLife. 2022 Mar 9;11:e76963. doi: 10.7554/eLife.76963 (PMC9007586; doi:10.7554/eLife.76963)

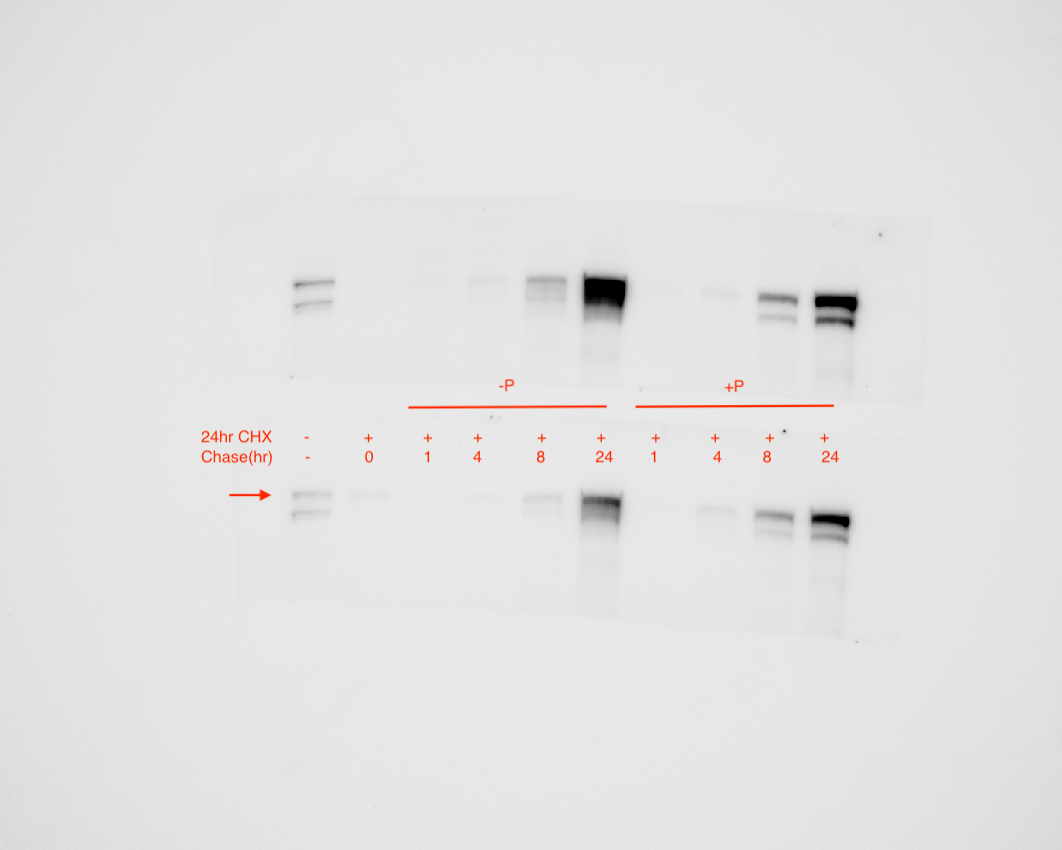

Supplement: Figure 3—source data 1. [file elife-76963-fig3-data1.zip › Figure 3-source data/Figure 3 C Western Blot Source Data/Figure 3C COL1A1.tif]

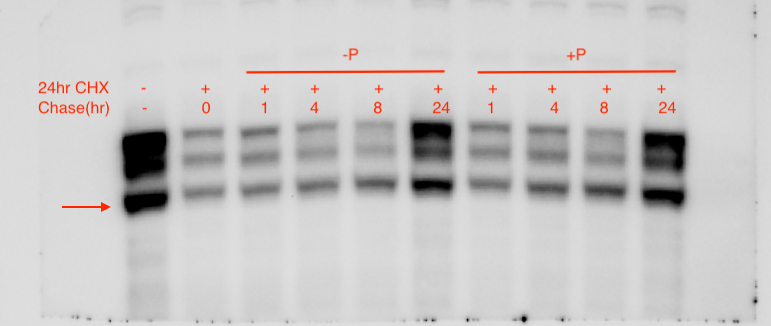

Supplement: Figure 3—source data 1. [file elife-76963-fig3-data1.zip › Figure 3-source data/Figure 3 C Western Blot Source Data/Figure 3C SMAD1.tiff]

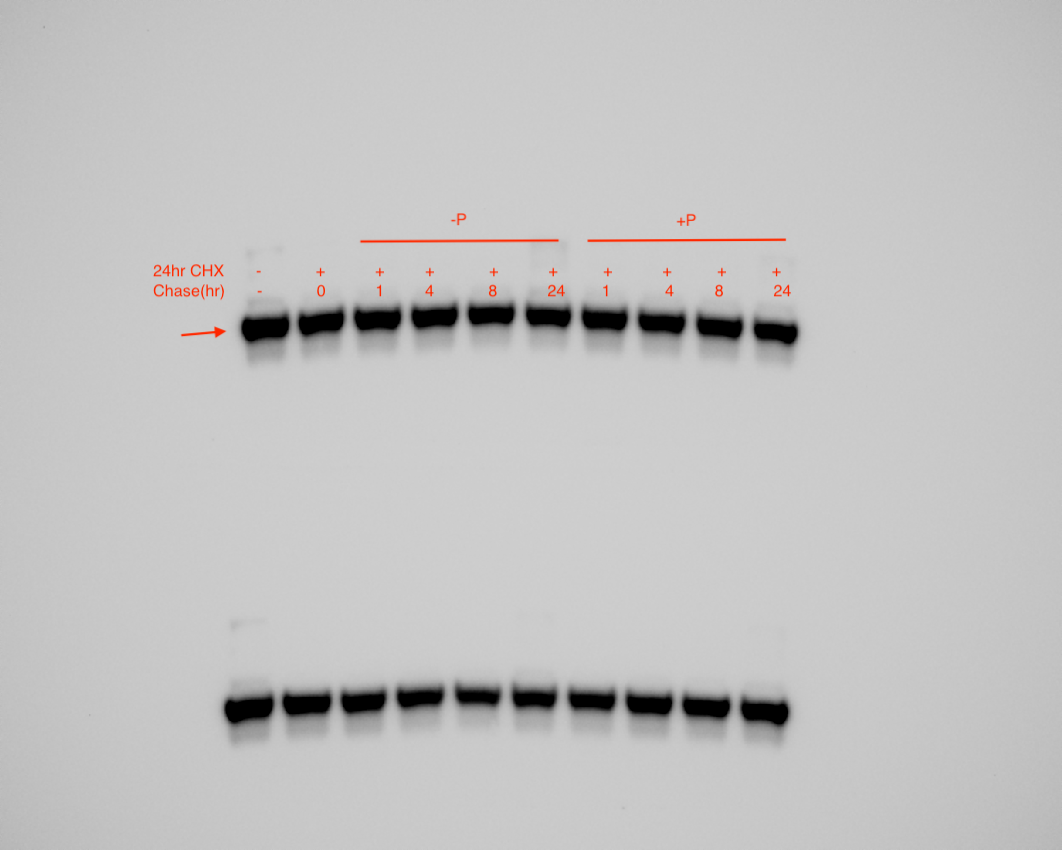

Supplement: Figure 3—source data 1. [file elife-76963-fig3-data1.zip › Figure 3-source data/Figure 3 C Western Blot Source Data/Figure 3C TUBA.tif]

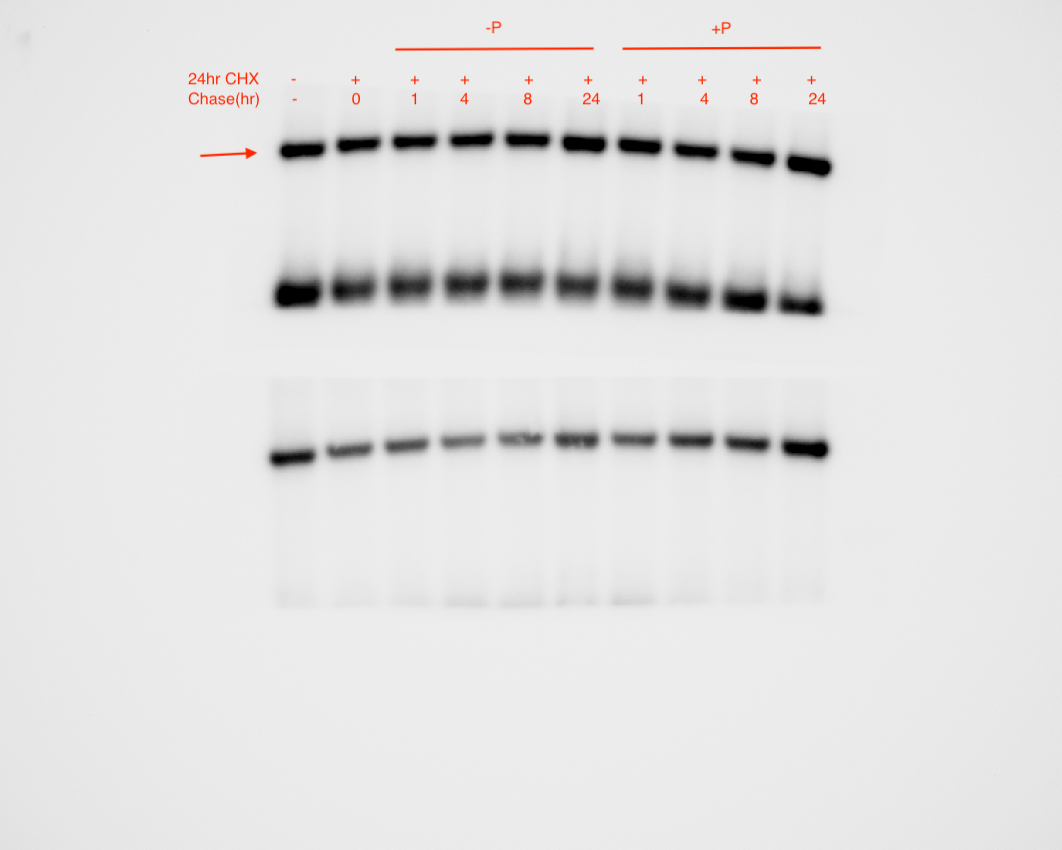

Supplement: Figure 3—source data 1. [file elife-76963-fig3-data1.zip › Figure 3-source data/Figure 3 C Western Blot Source Data/Figure 3C S6.tif]

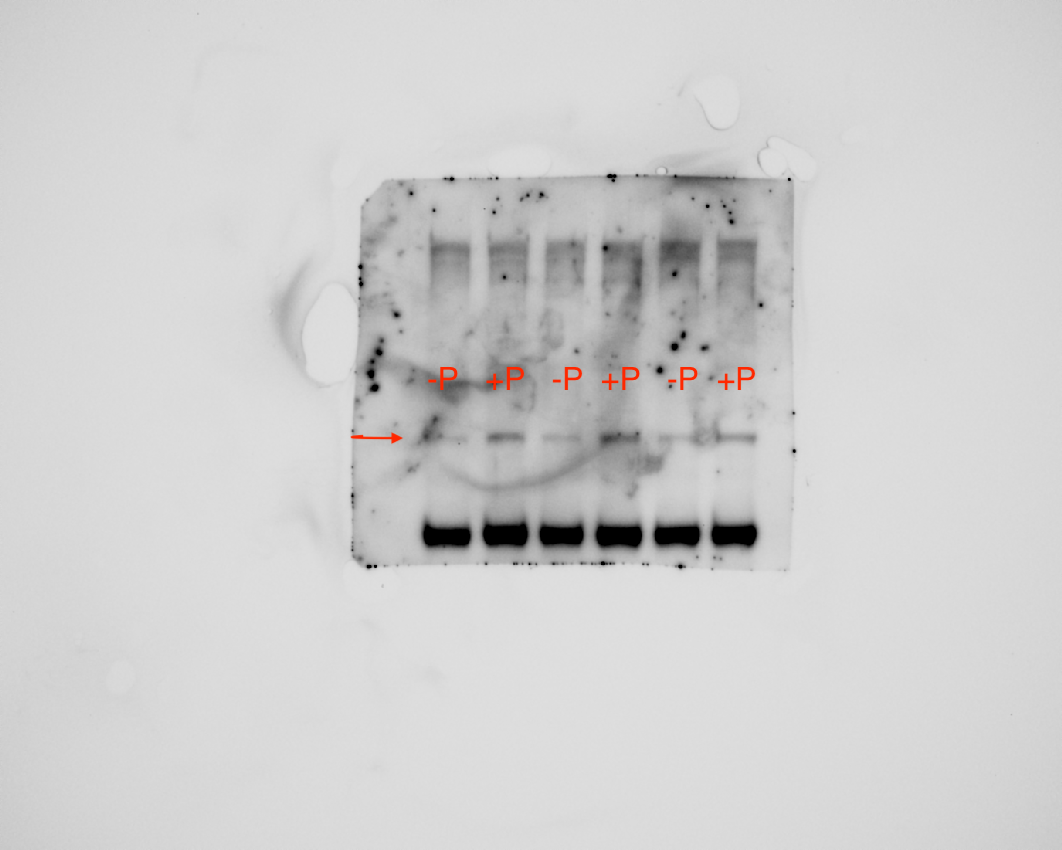

Supplement: Figure 3—source data 1. [file elife-76963-fig3-data1.zip › Figure 3-source data/Figure 3 A Western Blot Source Data/Figure 3A ATF4.tif]

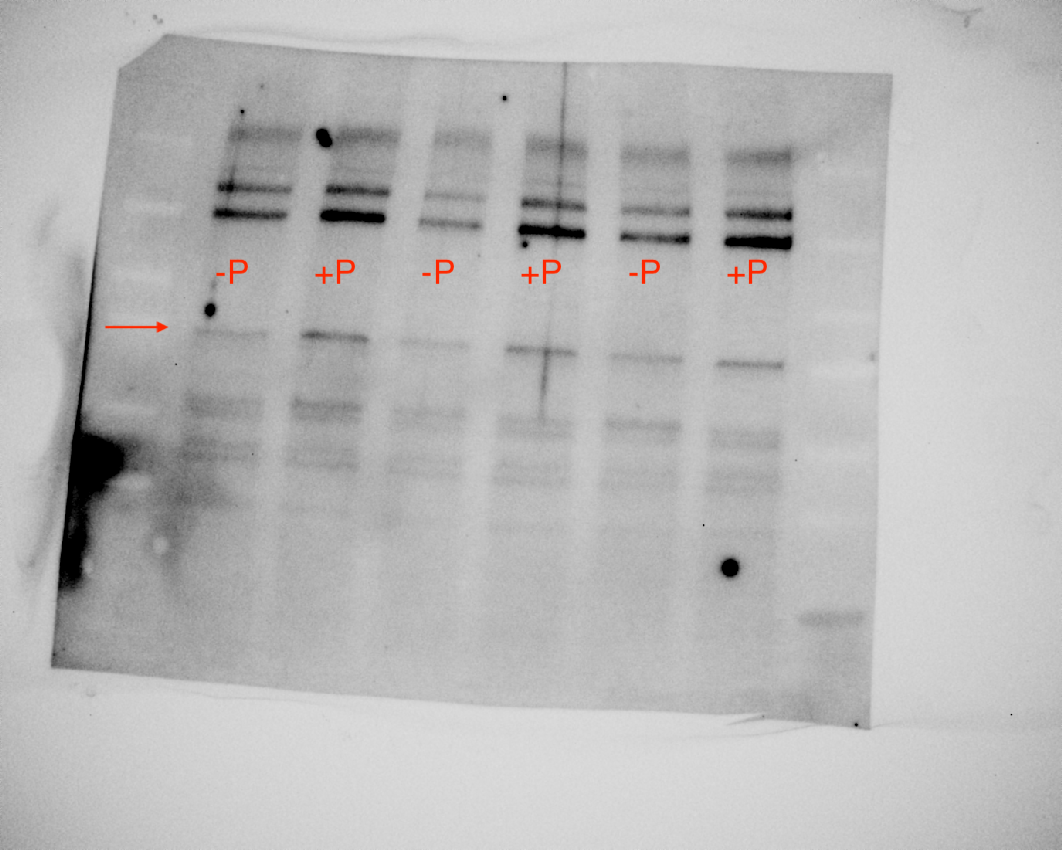

Supplement: Figure 3—source data 1. [file elife-76963-fig3-data1.zip › Figure 3-source data/Figure 3 A Western Blot Source Data/Figure 3A OSX.tif]

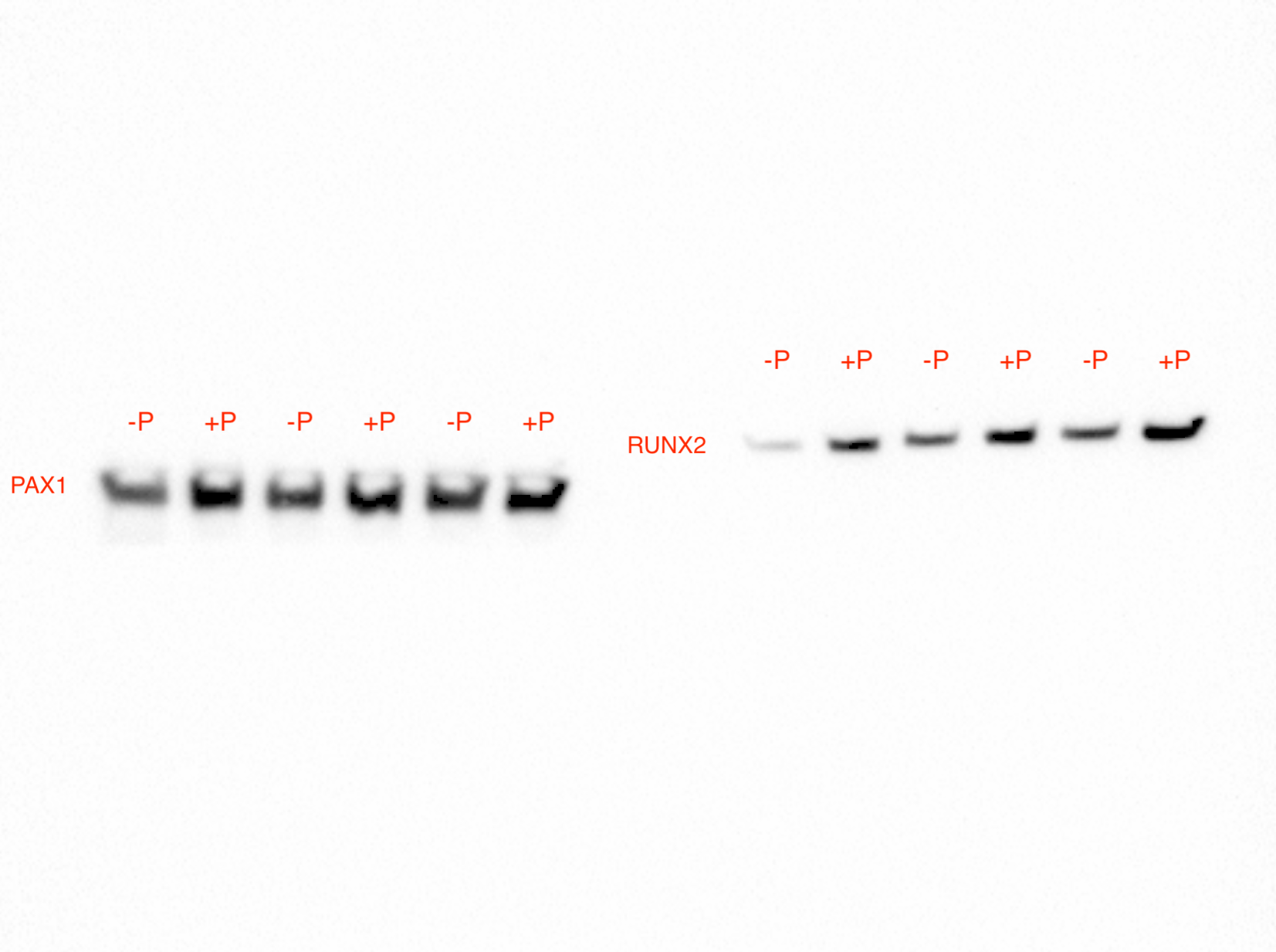

Supplement: Figure 3—source data 1. [file elife-76963-fig3-data1.zip › Figure 3-source data/Figure 3 A Western Blot Source Data/Figure 3A RUNX2 and PAX1.tif]

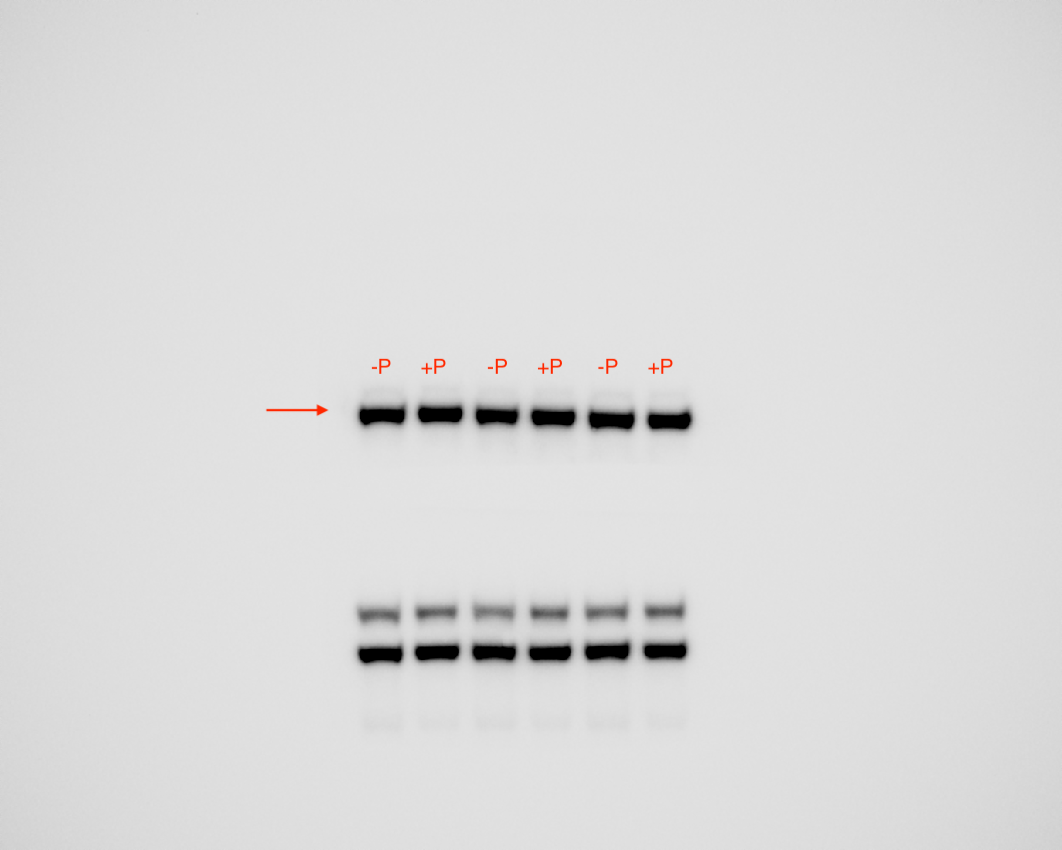

Supplement: Figure 3—source data 1. [file elife-76963-fig3-data1.zip › Figure 3-source data/Figure 3 A Western Blot Source Data/Figure 3A TUBA.tif]

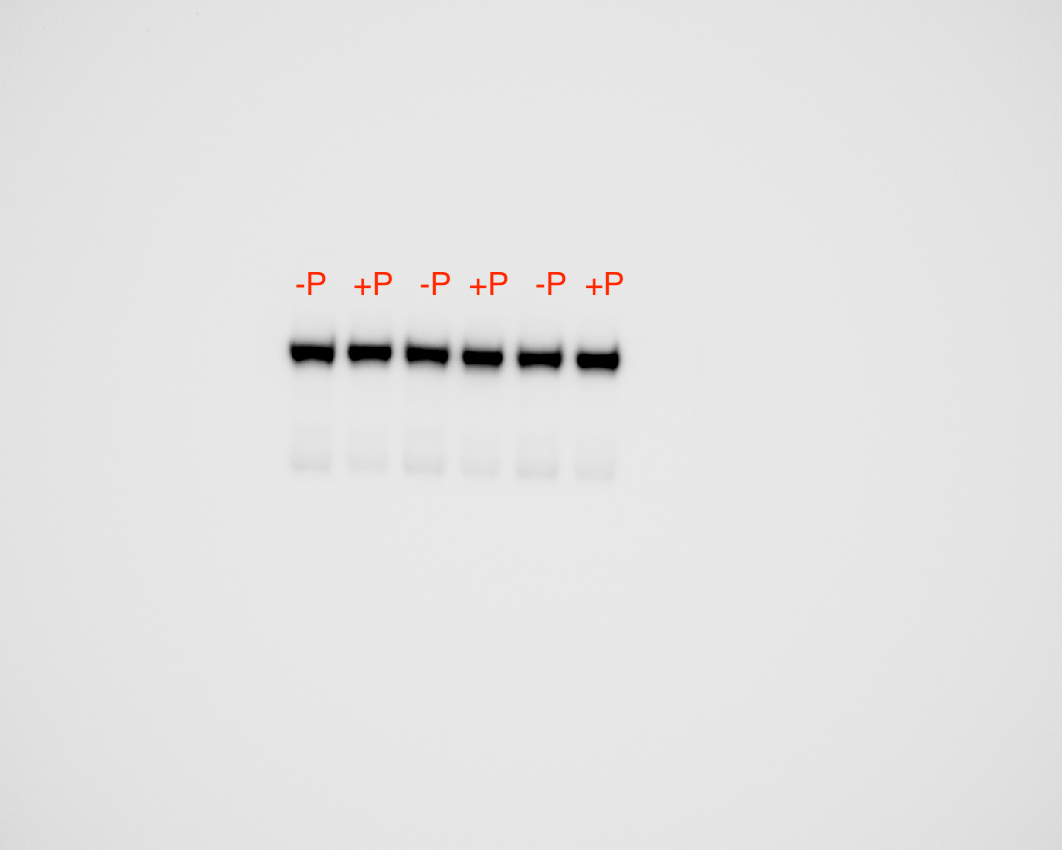

Supplement: Figure 3—source data 1. [file elife-76963-fig3-data1.zip › Figure 3-source data/Figure 3 A Western Blot Source Data/Figure 3A AKT.tif]

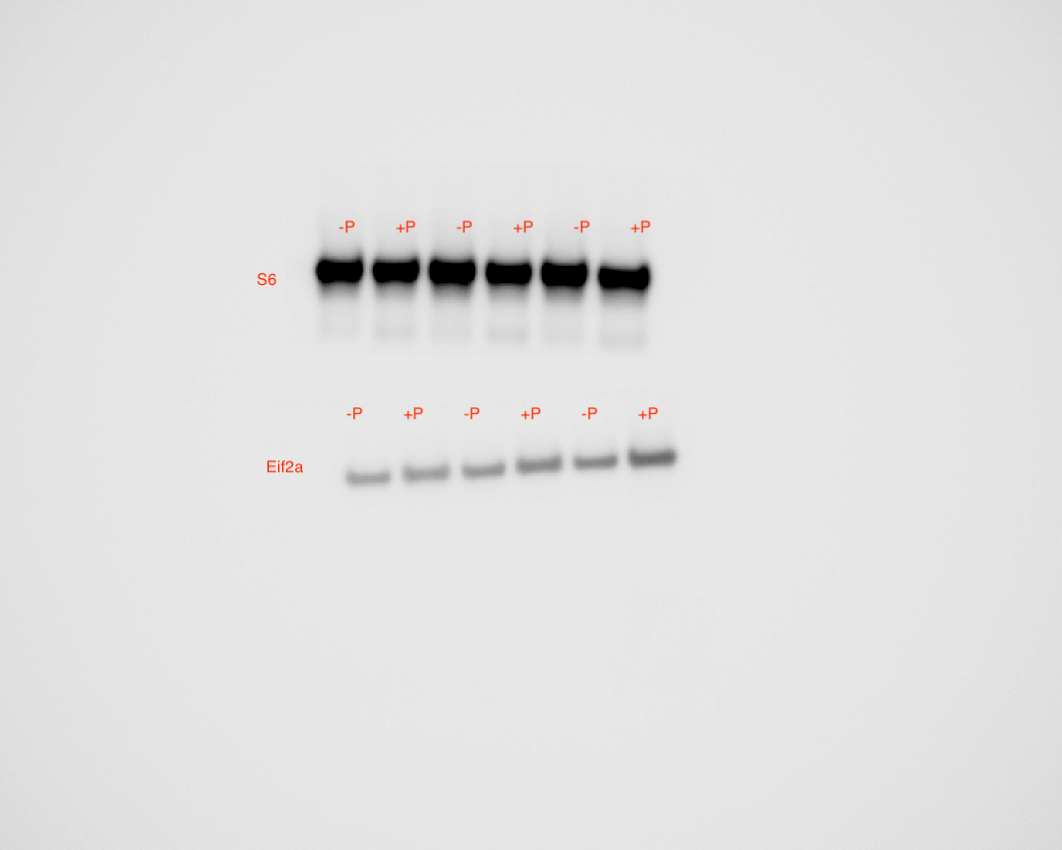

Supplement: Figure 3—source data 1. [file elife-76963-fig3-data1.zip › Figure 3-source data/Figure 3 A Western Blot Source Data/Figure 3A S6 Top EIF2A Bottom.tiff]

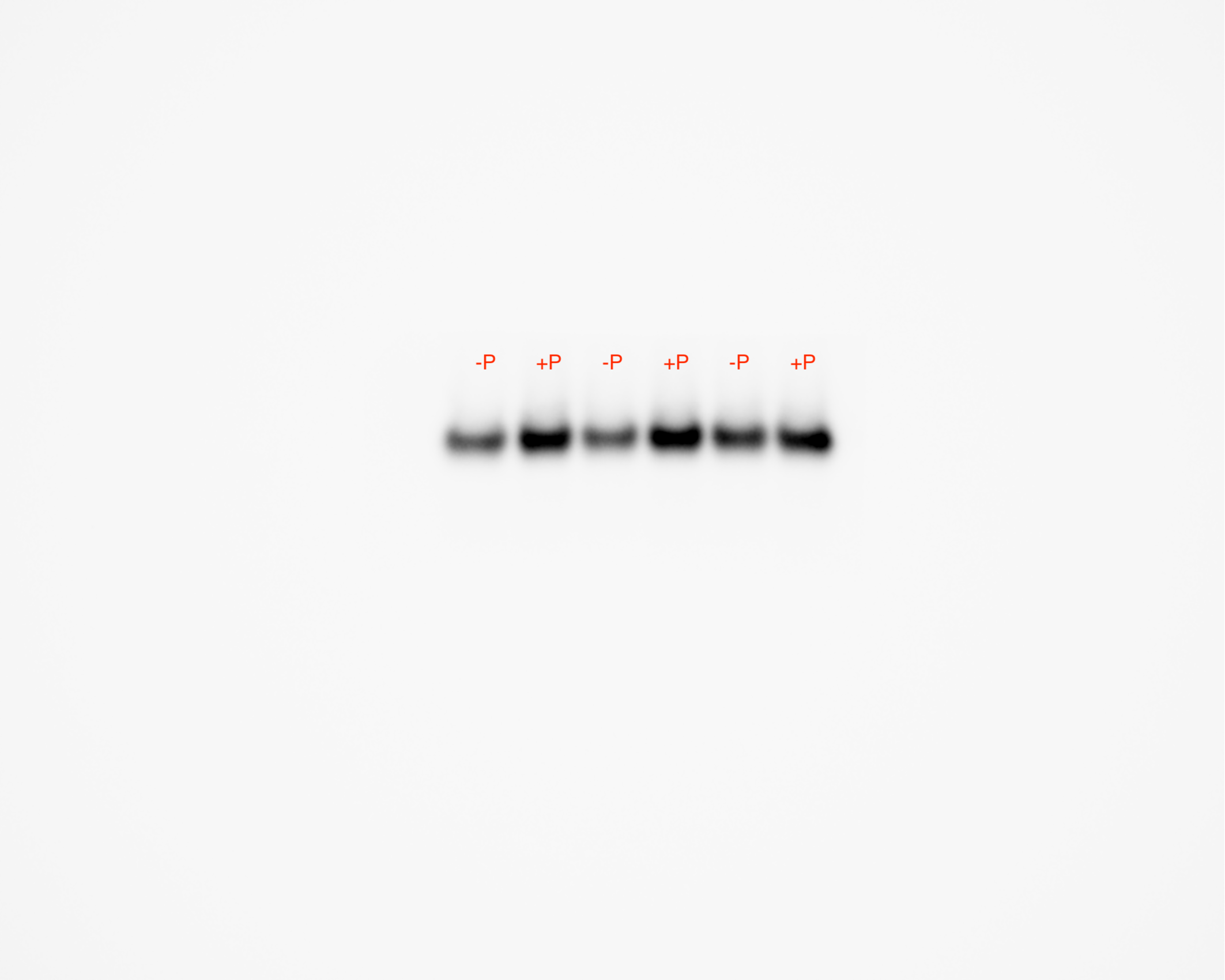

Supplement: Figure 3—source data 1. [file elife-76963-fig3-data1.zip › Figure 3-source data/Figure 3 A Western Blot Source Data/Figure 3A EIF4EBP1.tif]

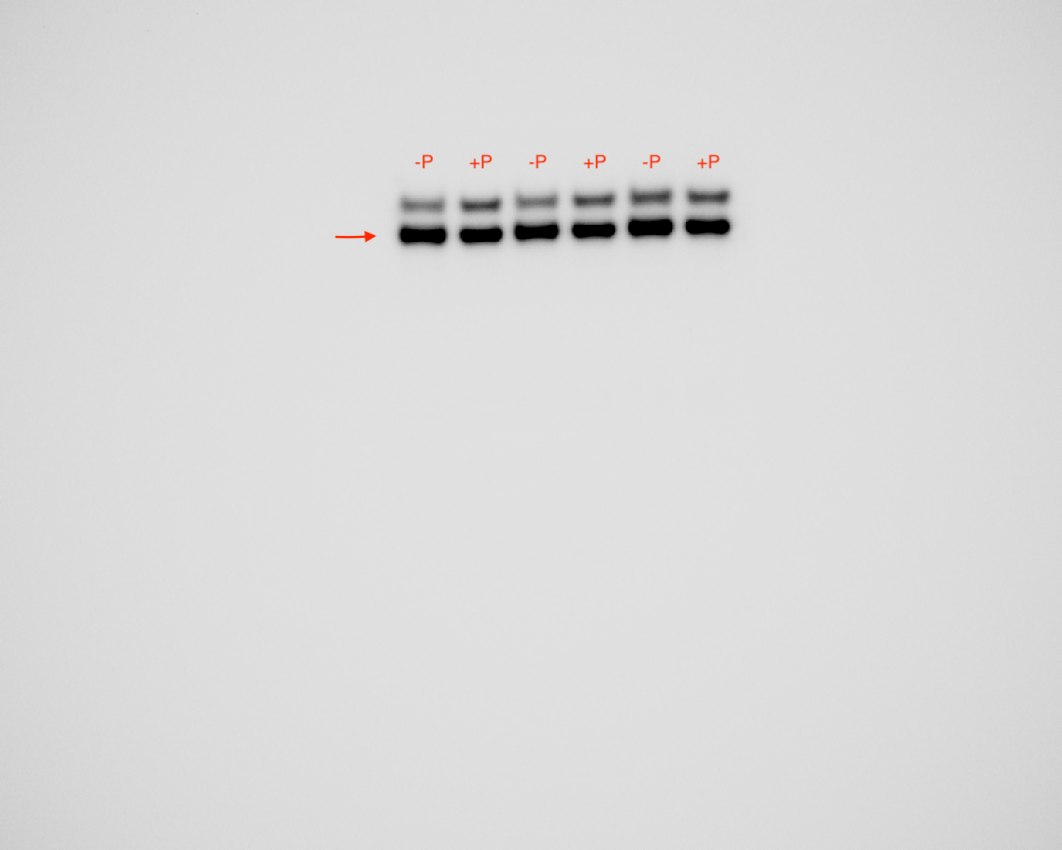

Supplement: Figure 3—source data 1. [file elife-76963-fig3-data1.zip › Figure 3-source data/Figure 3 A Western Blot Source Data/Figure 3A ERK.tif]

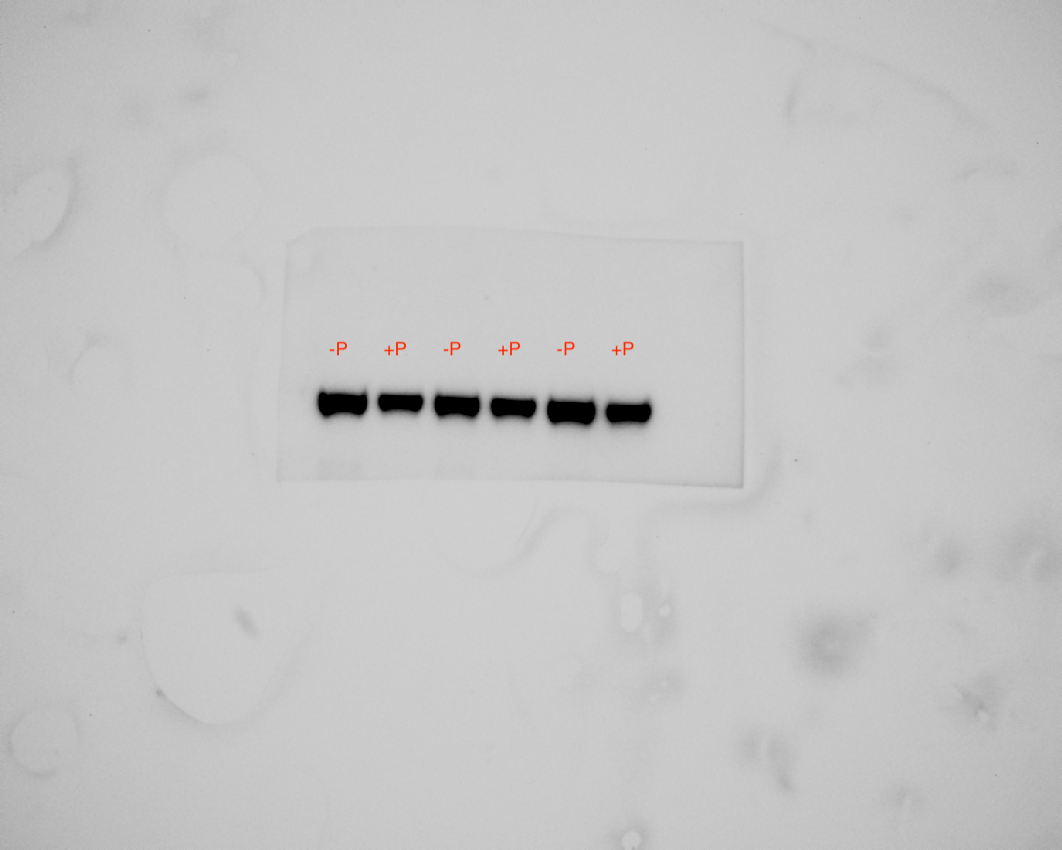

Supplement: Figure 3—source data 1. [file elife-76963-fig3-data1.zip › Figure 3-source data/Figure 3 A Western Blot Source Data/Figure 3A PHGDH.tif]

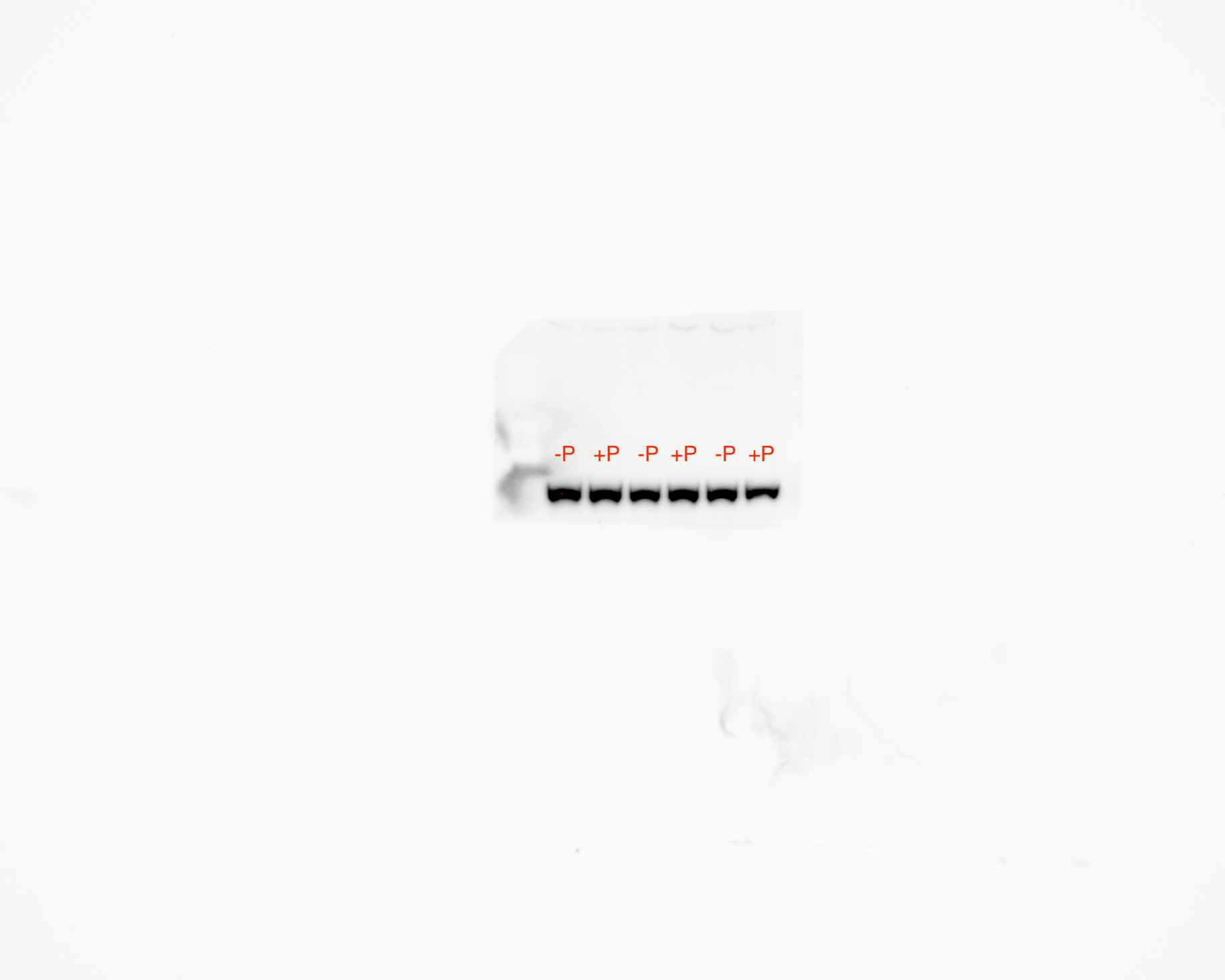

Supplement: Figure 3—source data 1. [file elife-76963-fig3-data1.zip › Figure 3-source data/Figure 3 A Western Blot Source Data/Figure 3A ACTB.tif]

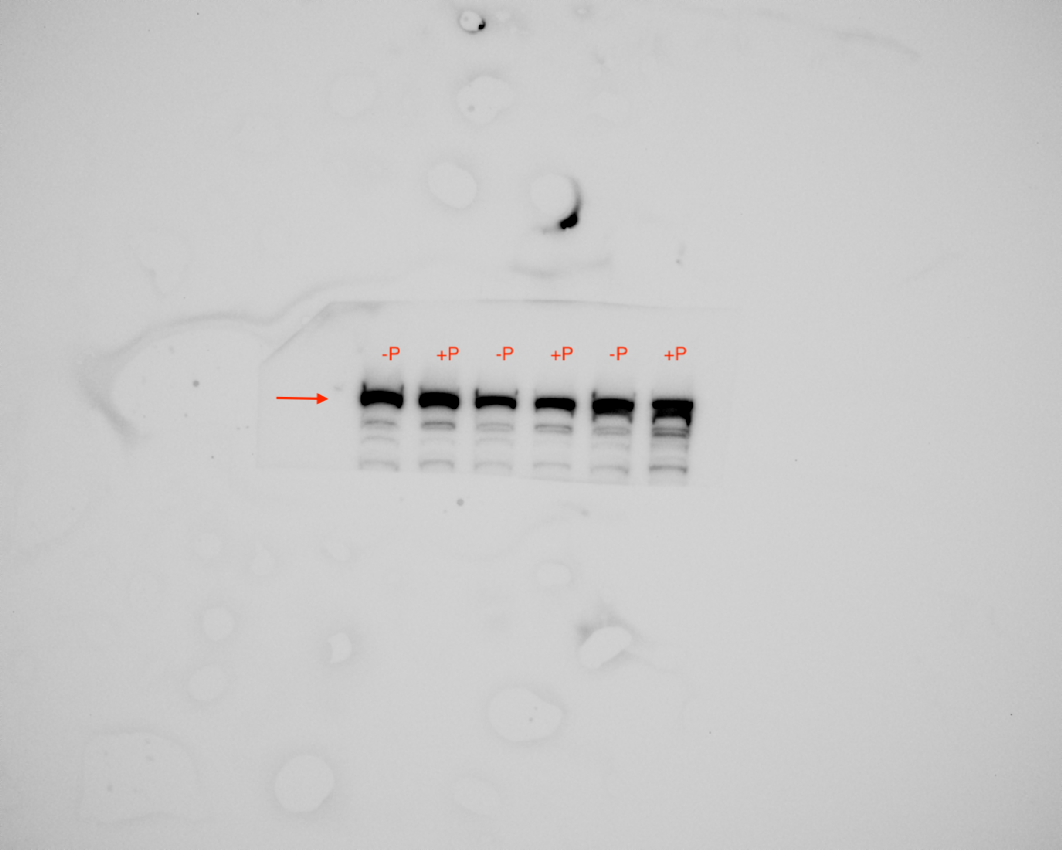

Supplement: Figure 3—source data 1. [file elife-76963-fig3-data1.zip › Figure 3-source data/Figure 3 A Western Blot Source Data/Figure 3A mTOR.tif]

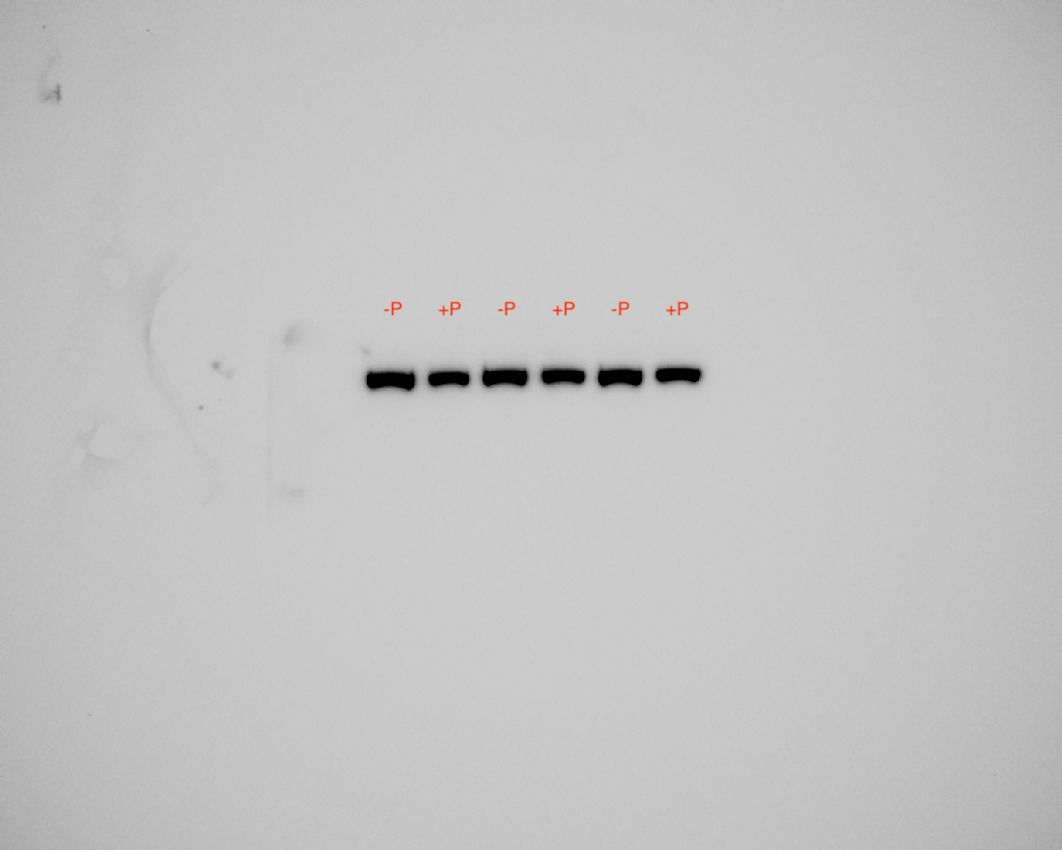

Supplement: Figure 3—source data 1. [file elife-76963-fig3-data1.zip › Figure 3-source data/Figure 3 A Western Blot Source Data/Figure 3A EEF2.tif]

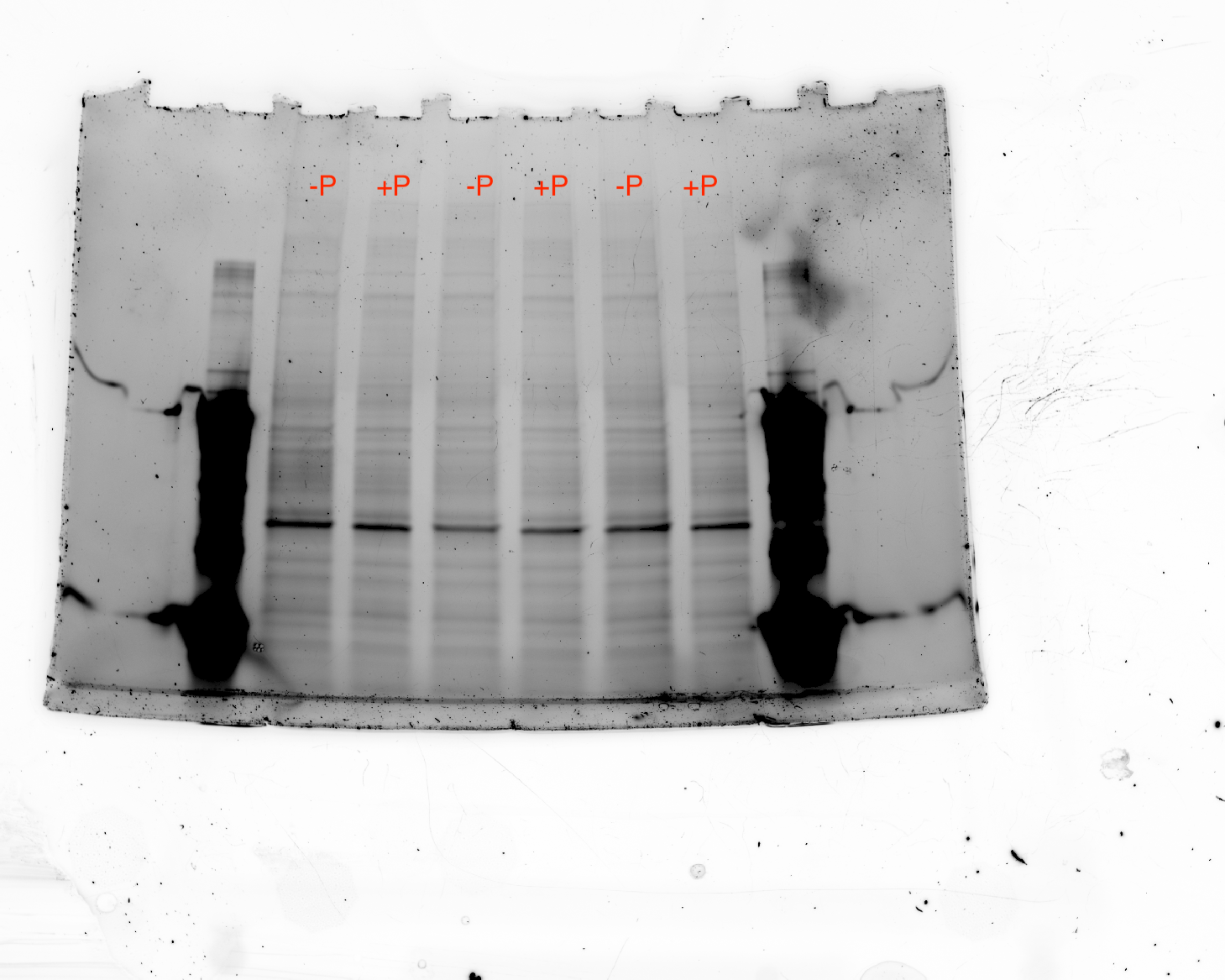

Supplement: Figure 3—source data 1. [file elife-76963-fig3-data1.zip › Figure 3-source data/Figure 3 A Western Blot Source Data/Figure 3A stainfree gel.tif]

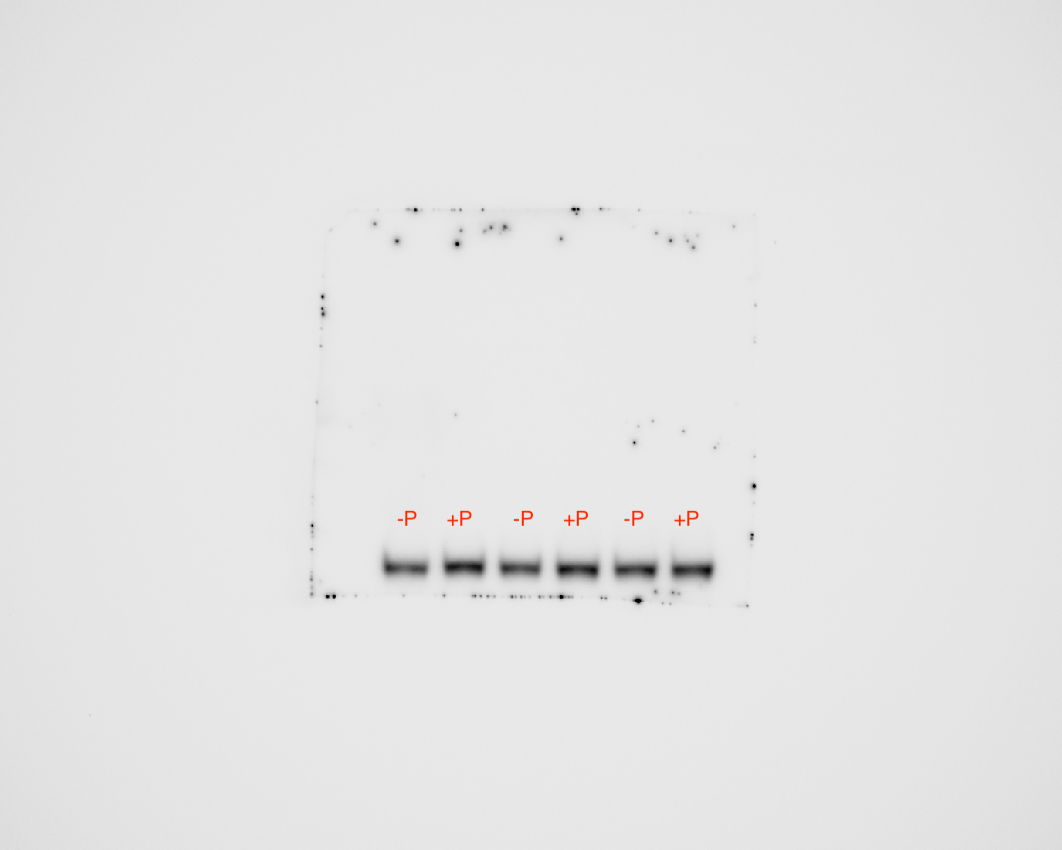

Supplement: Figure 3—source data 1. [file elife-76963-fig3-data1.zip › Figure 3-source data/Figure 3 A Western Blot Source Data/Figure 3A SMAD1.tif]

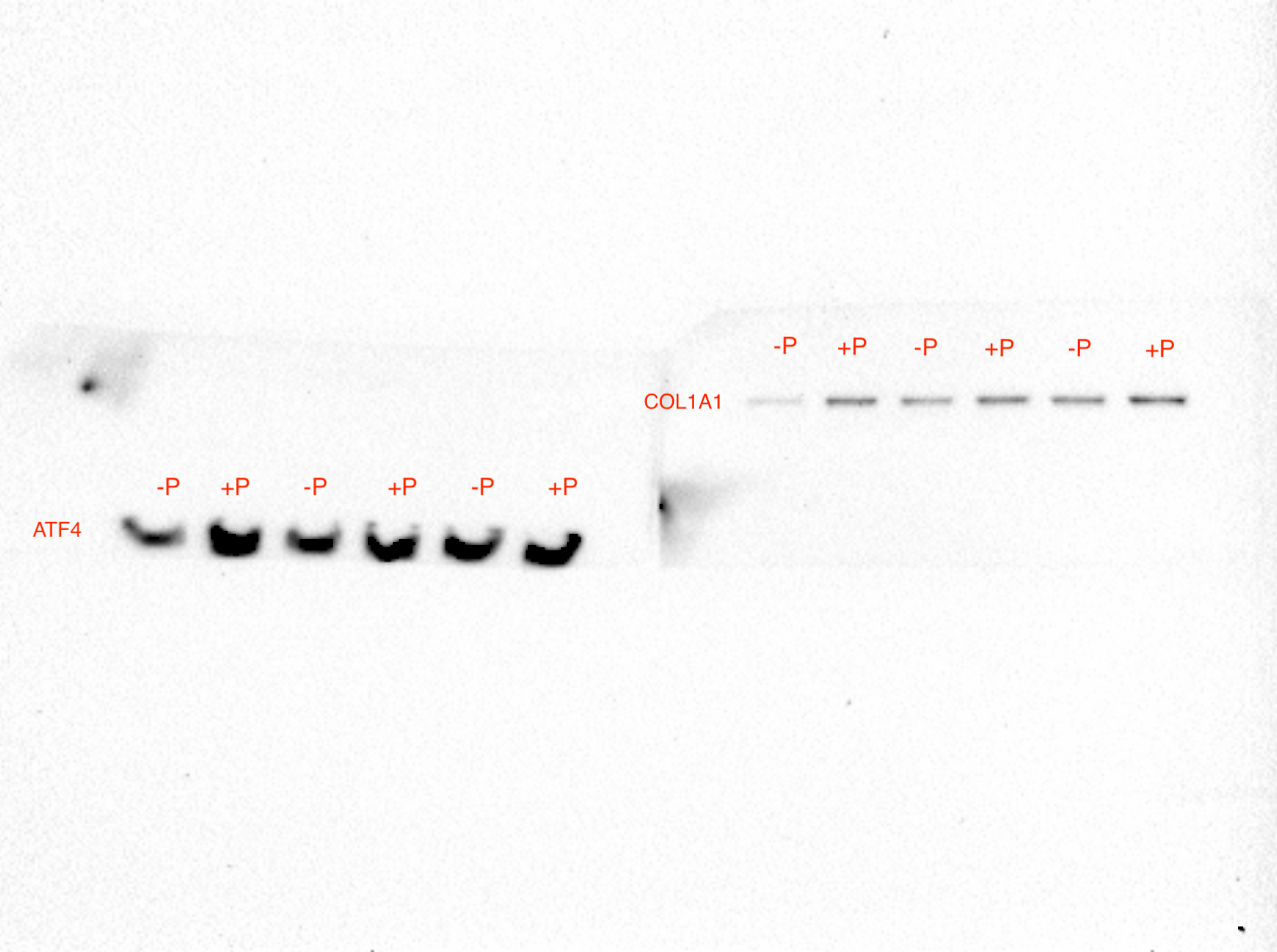

Supplement: Figure 3—source data 1. [file elife-76963-fig3-data1.zip › Figure 3-source data/Figure 3 A Western Blot Source Data/Figure 3A COL1A1 and ATF2.tif]

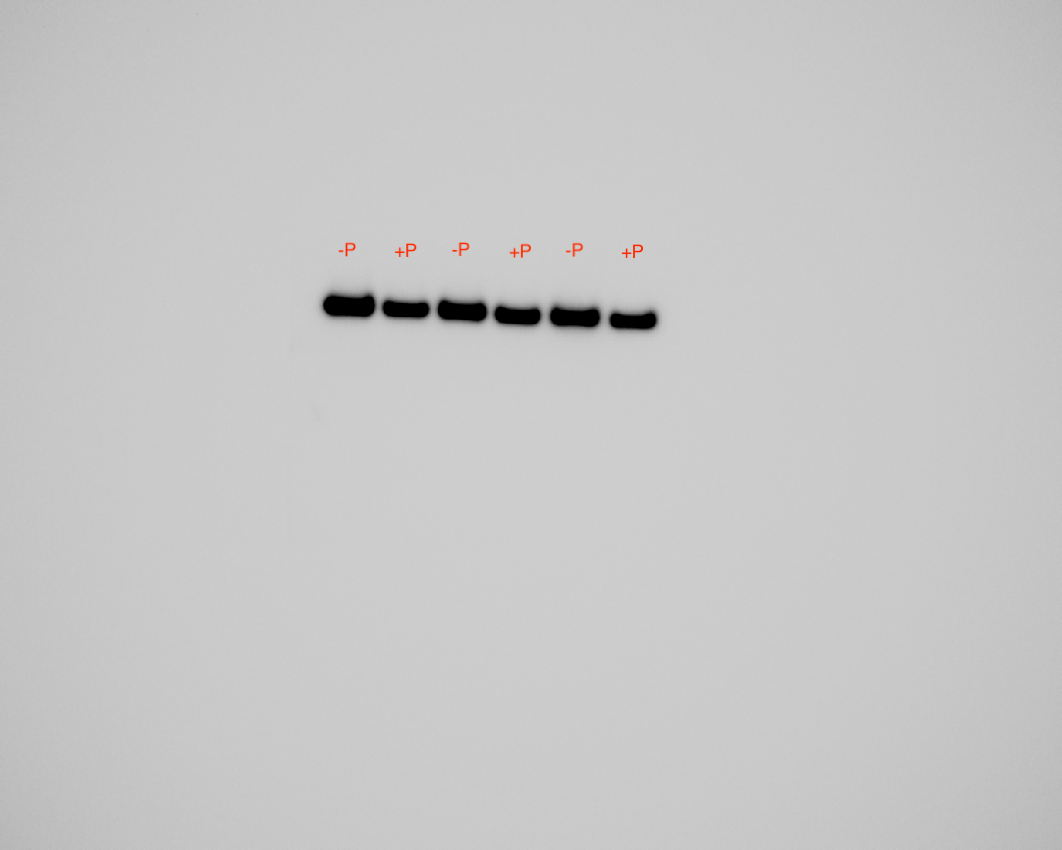

Supplement: Figure 3—figure supplement 1—source data 1. [file elife-76963-fig3-figsupp1-data1.zip › Figure 3 Supplement-source data/Figure 3 Supplement 1B Western Blot Source Data/Figure 3 Supplement 1B pS6.tif]

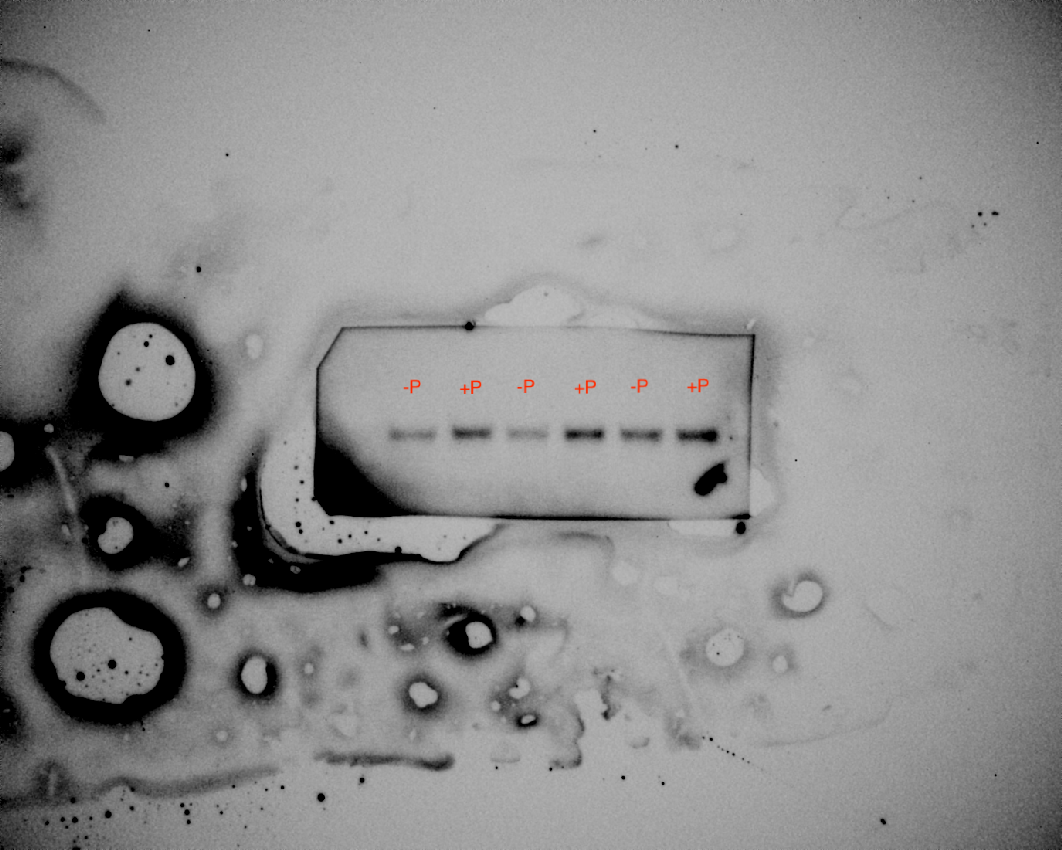

Supplement: Figure 3—figure supplement 1—source data 1. [file elife-76963-fig3-figsupp1-data1.zip › Figure 3 Supplement-source data/Figure 3 Supplement 1B Western Blot Source Data/Figure 3 Supplement 1B pEIF2A.tif]

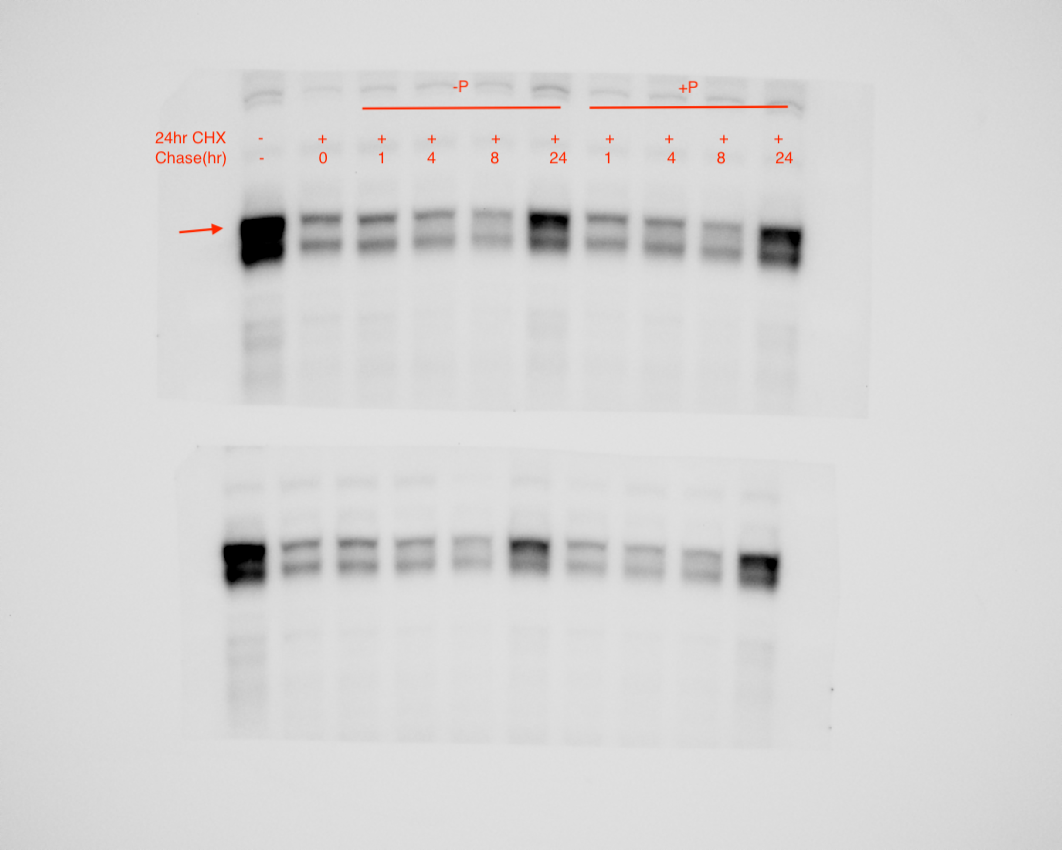

Supplement: Figure 3—figure supplement 1—source data 1. [file elife-76963-fig3-figsupp1-data1.zip › Figure 3 Supplement-source data/Figure 3 Supplement 1D Western Blot Source Data/Figure 3 Supplement 1D Runx2.tif]

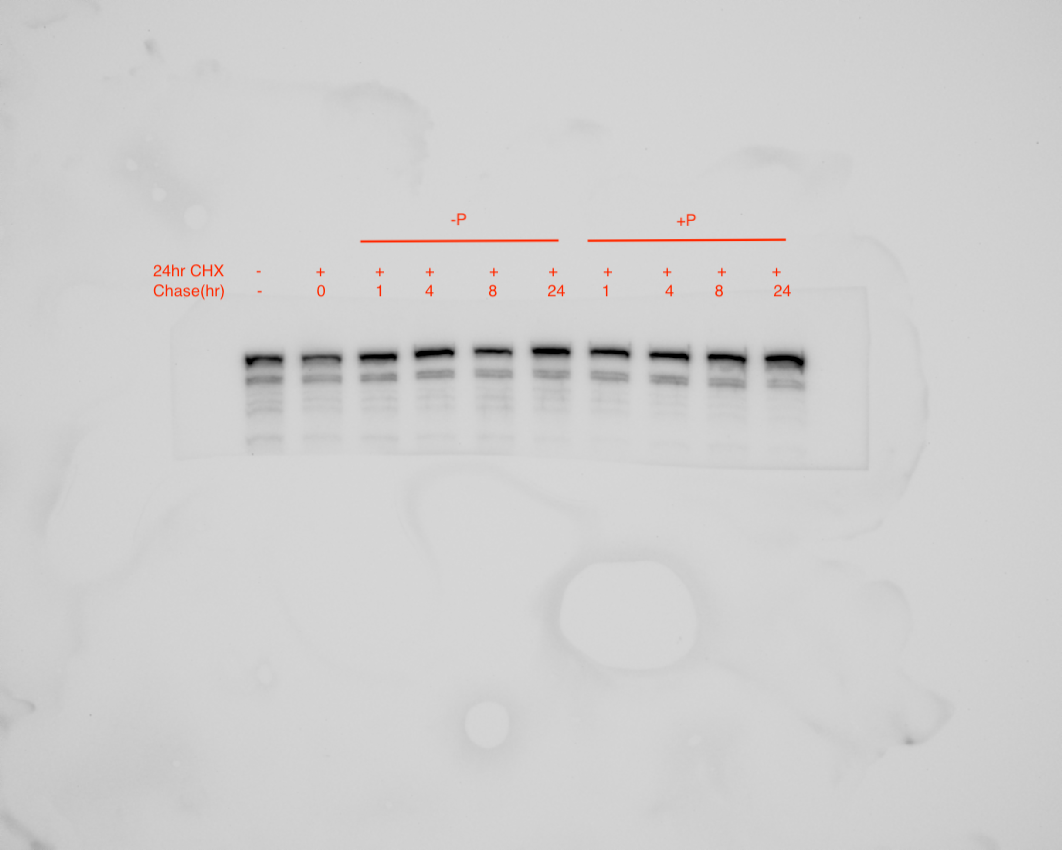

Supplement: Figure 3—figure supplement 1—source data 1. [file elife-76963-fig3-figsupp1-data1.zip › Figure 3 Supplement-source data/Figure 3 Supplement 1D Western Blot Source Data/Figure 3 Supplement 1D mTOR.tif]

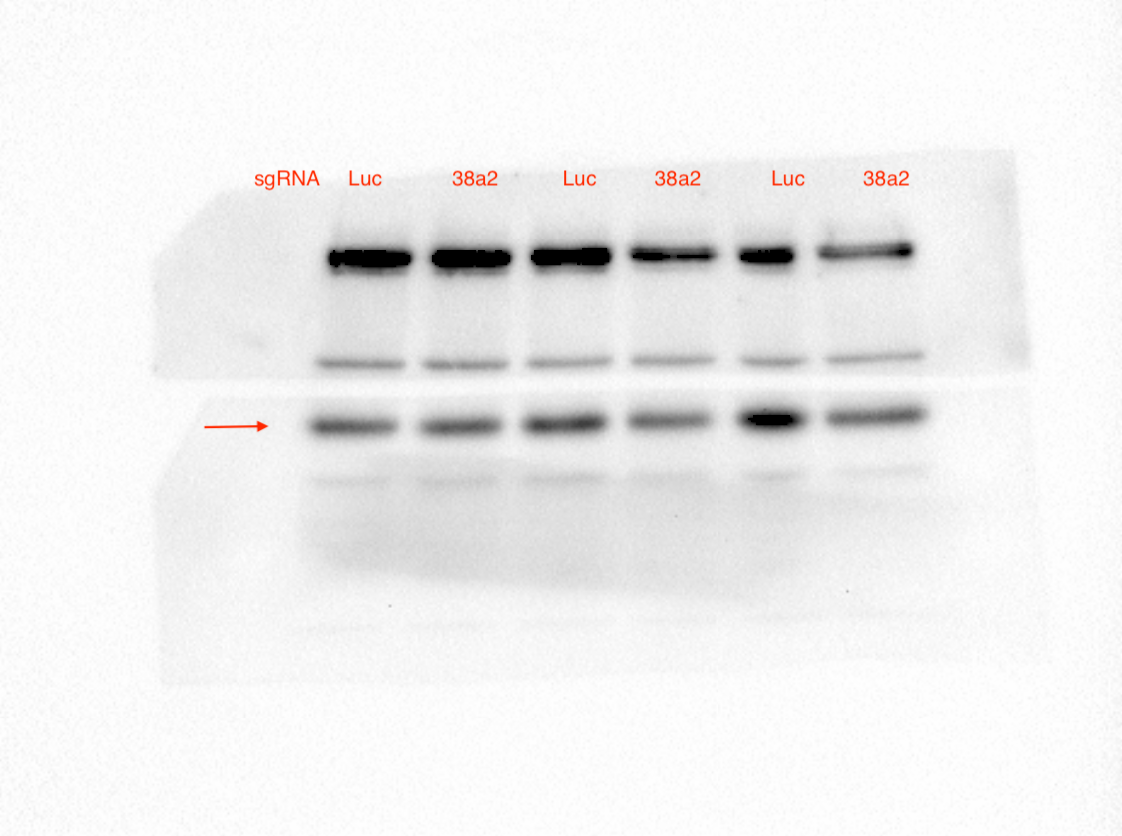

Supplement: Figure 4—source data 1. [file elife-76963-fig4-data1.zip › Figure 4-source data/Figure 4 C Western Blot Source Data/Figure 4C RUNX2 bottom.tif]

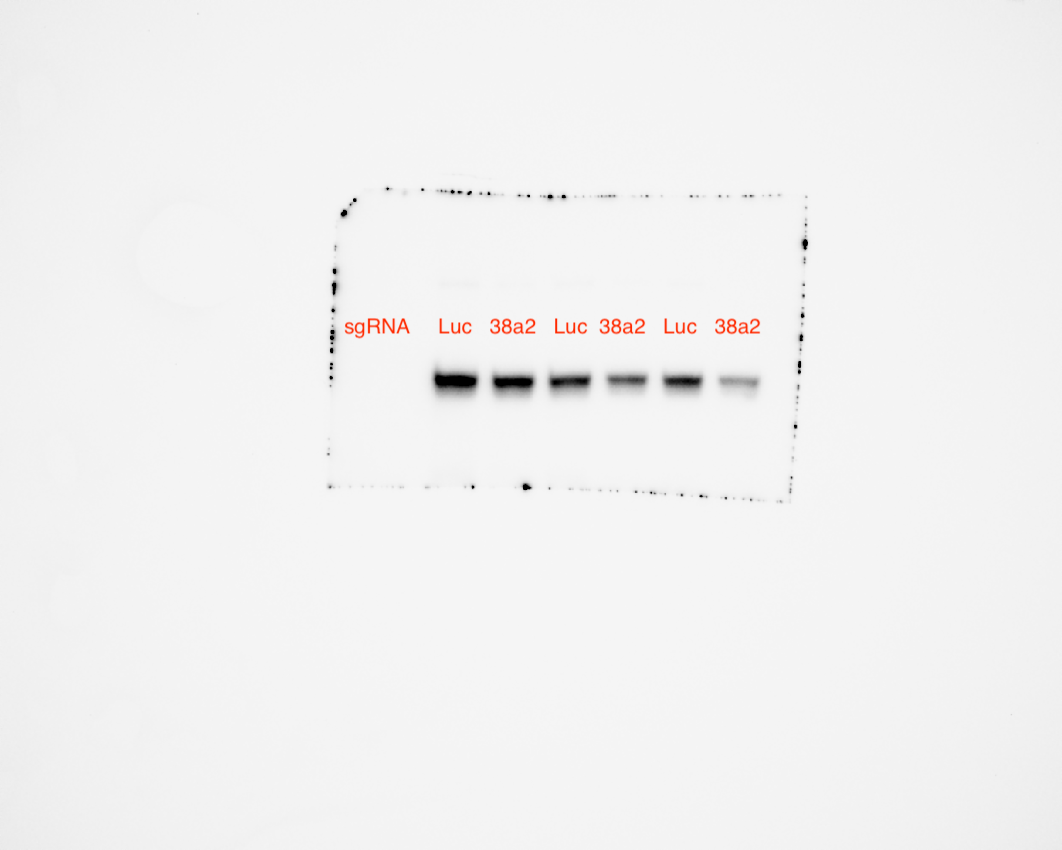

Supplement: Figure 4—source data 1. [file elife-76963-fig4-data1.zip › Figure 4-source data/Figure 4 C Western Blot Source Data/Figure 4C SMAD1.tif]

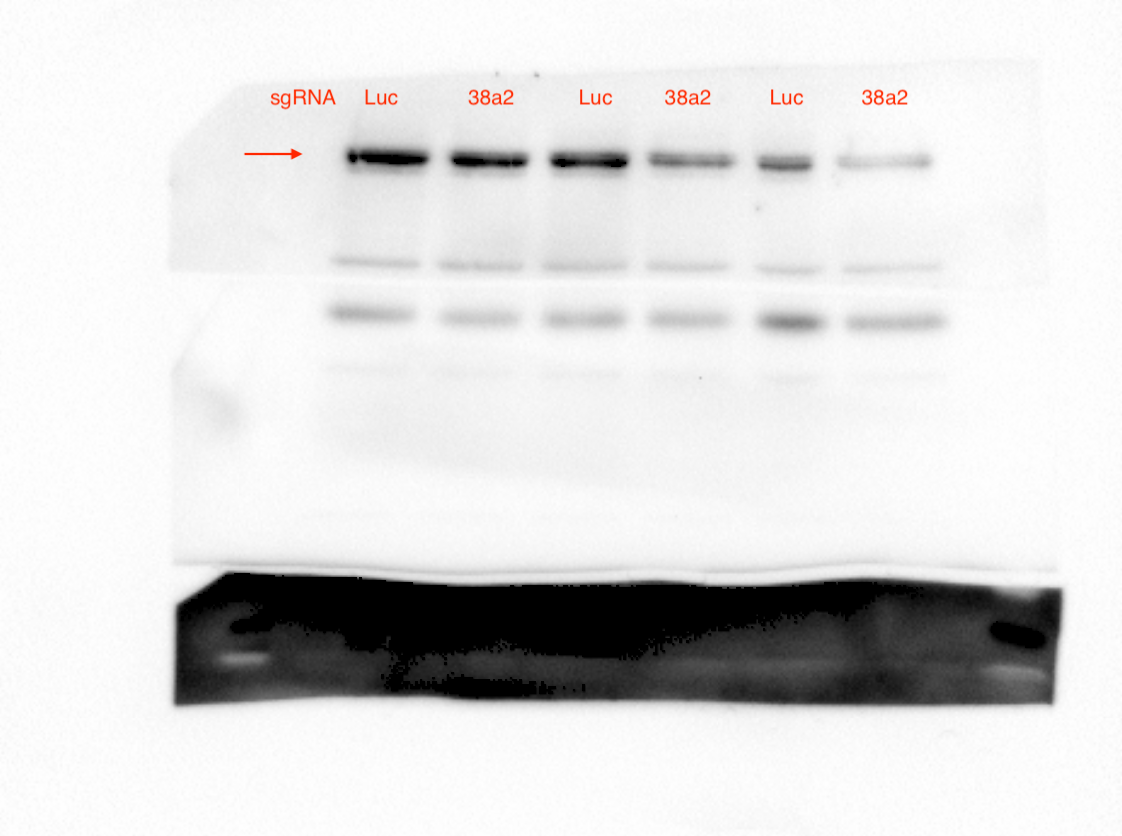

Supplement: Figure 4—source data 1. [file elife-76963-fig4-data1.zip › Figure 4-source data/Figure 4 C Western Blot Source Data/Figure 4C COL1A1 top.tif]

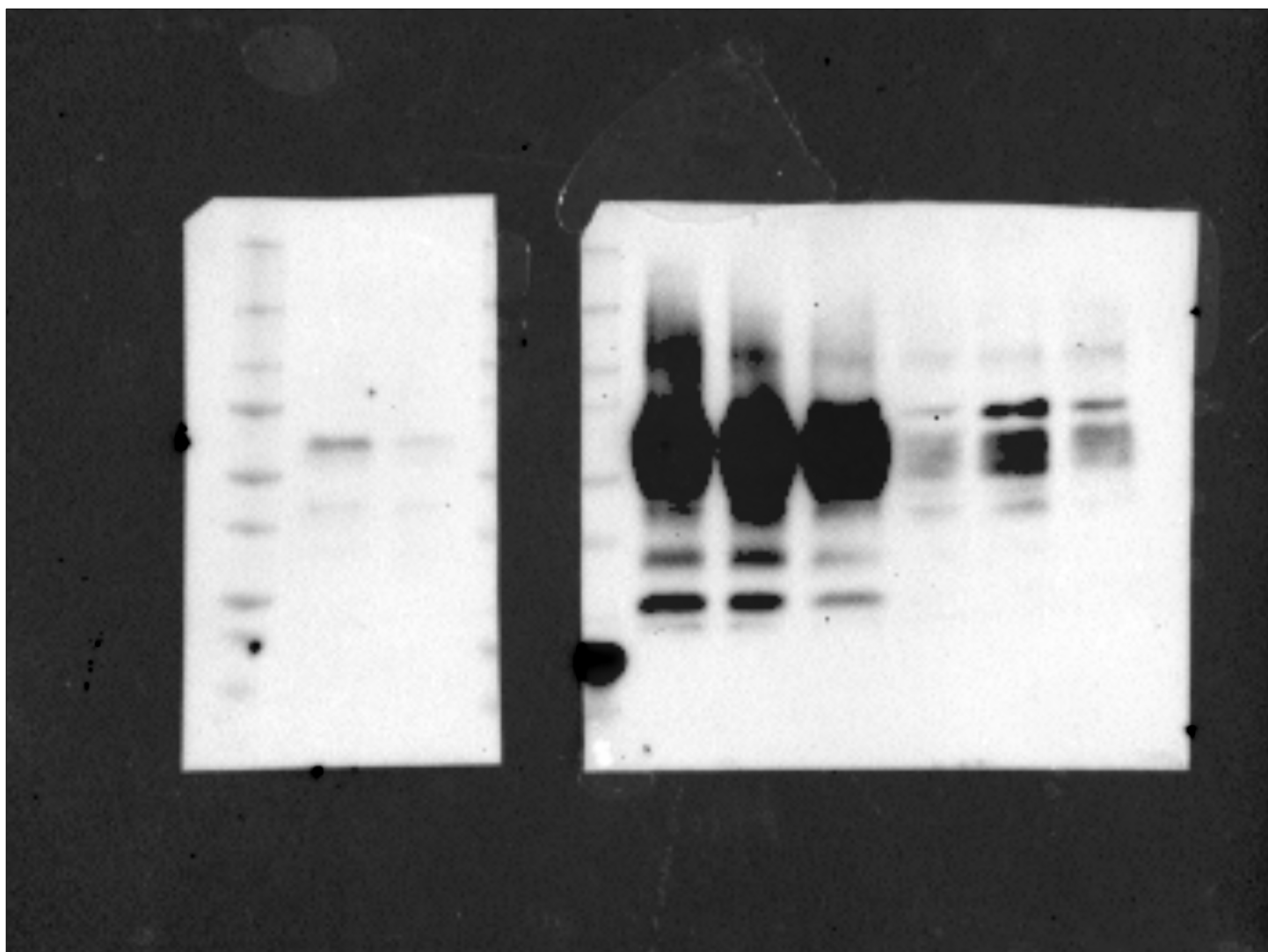

Supplement: Figure 4—source data 1. [file elife-76963-fig4-data1.zip › Figure 4-source data/Figure 4 C Western Blot Source Data/Figure 4C SNAT2.pdf]

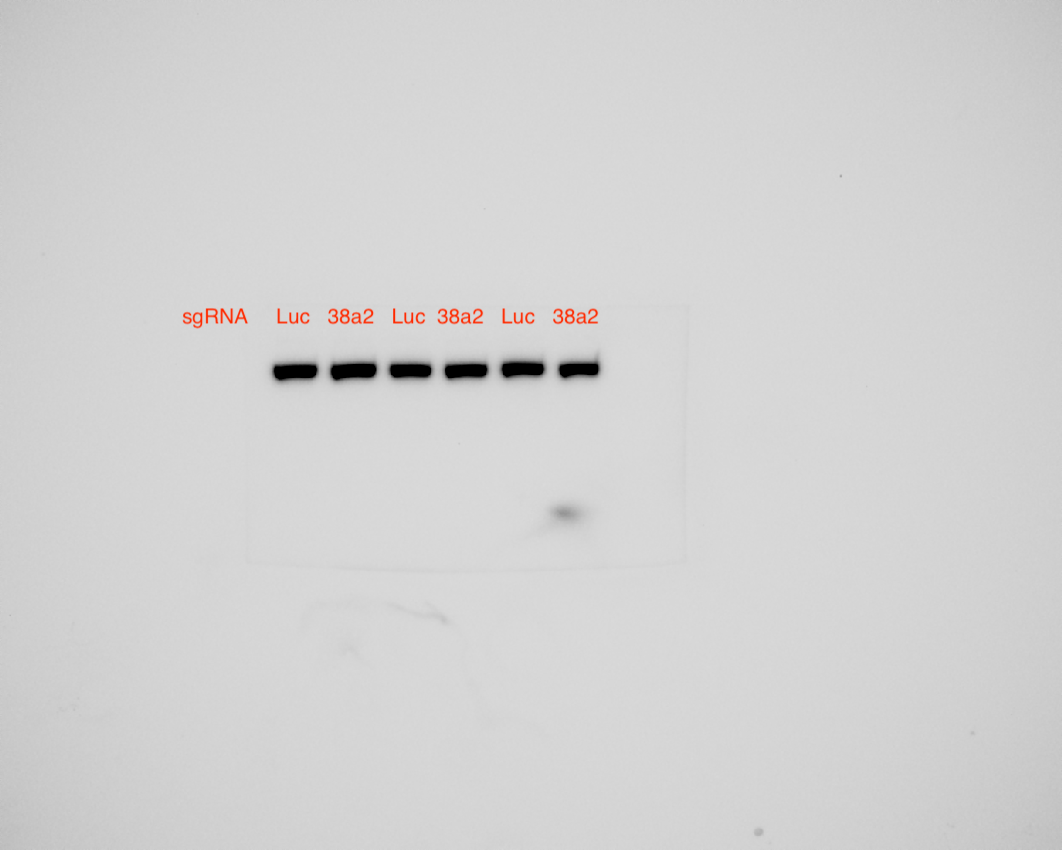

Supplement: Figure 4—source data 1. [file elife-76963-fig4-data1.zip › Figure 4-source data/Figure 4 C Western Blot Source Data/Figure 4C EEF2.tif]

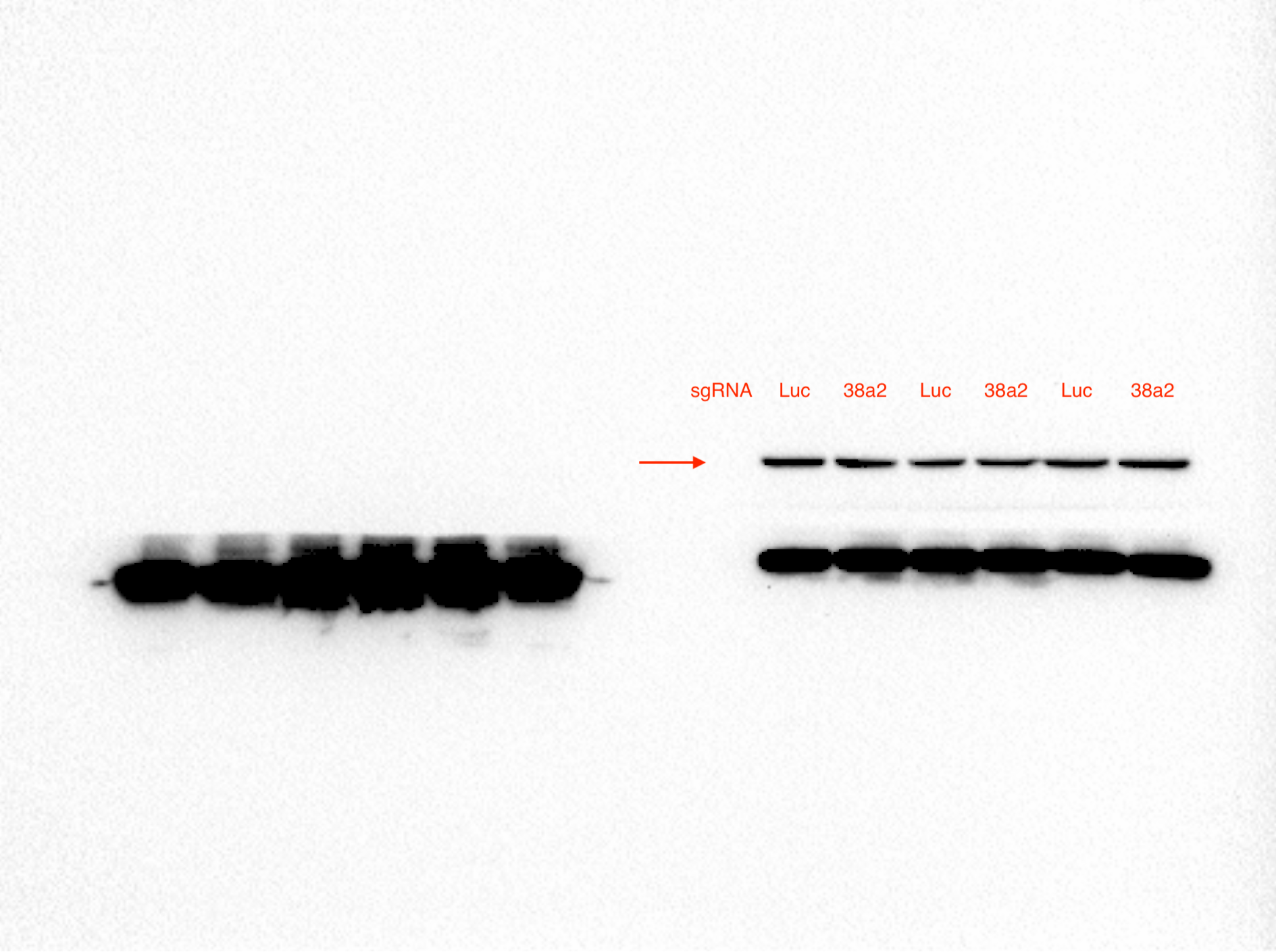

Supplement: Figure 4—source data 1. [file elife-76963-fig4-data1.zip › Figure 4-source data/Figure 4 C Western Blot Source Data/Figure 4C ACTB.tif]

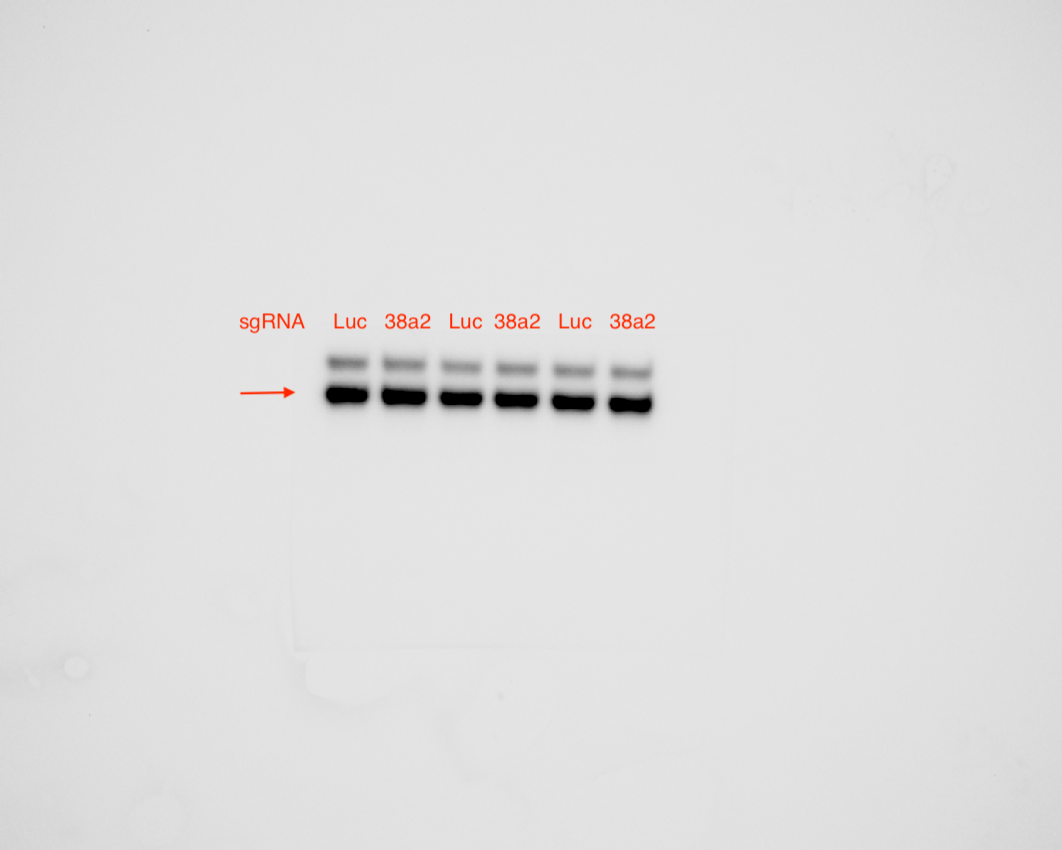

Supplement: Figure 4—source data 1. [file elife-76963-fig4-data1.zip › Figure 4-source data/Figure 4 C Western Blot Source Data/Figure 4C ERK.tif]

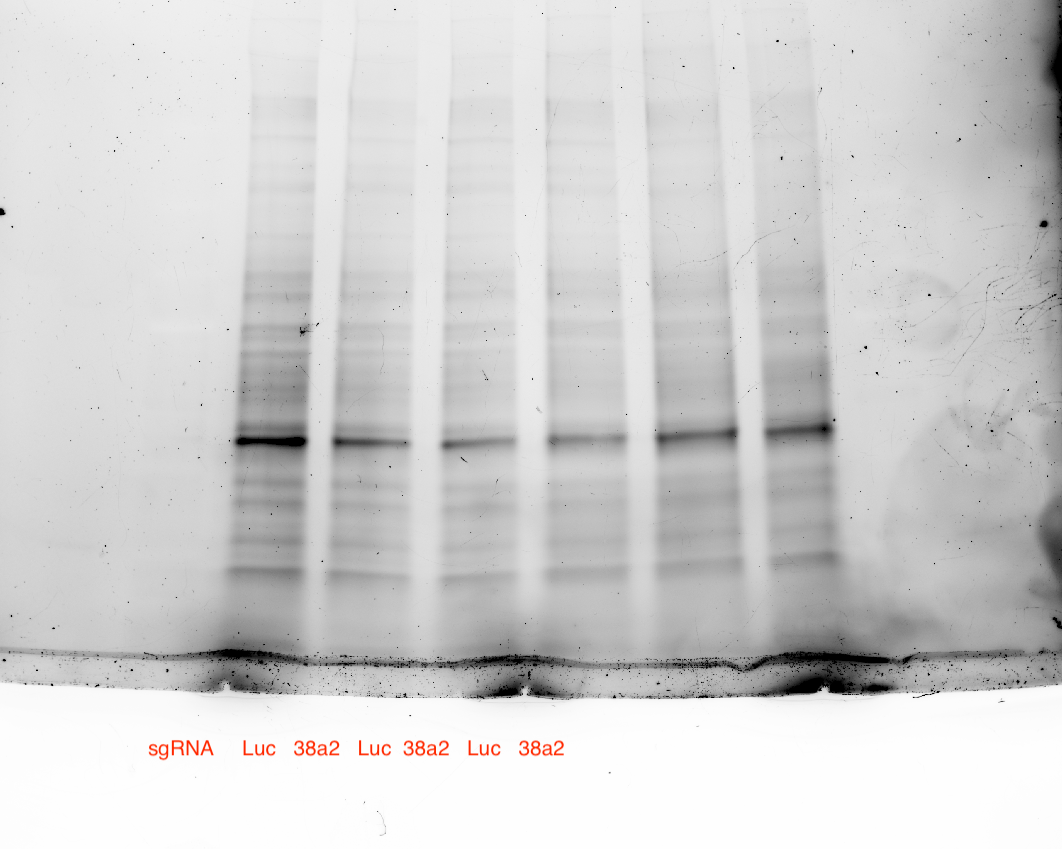

Supplement: Figure 4—source data 1. [file elife-76963-fig4-data1.zip › Figure 4-source data/Figure 4 C Western Blot Source Data/Figure 4C Stain-free gel.tif]

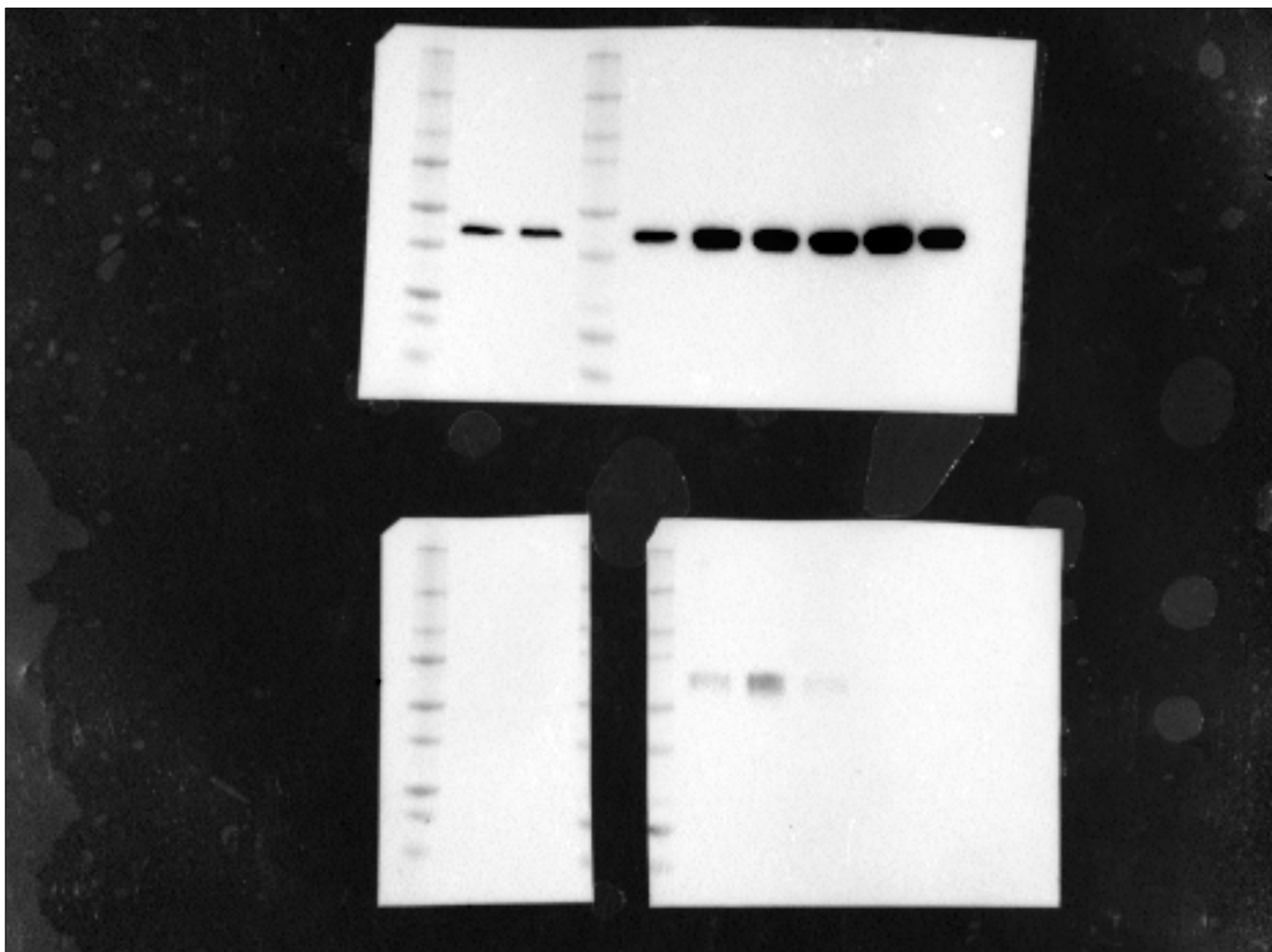

Supplement: Figure 4—source data 1. [file elife-76963-fig4-data1.zip › Figure 4-source data/Figure 4 C Western Blot Source Data/Figure 4C TUBA.pdf]

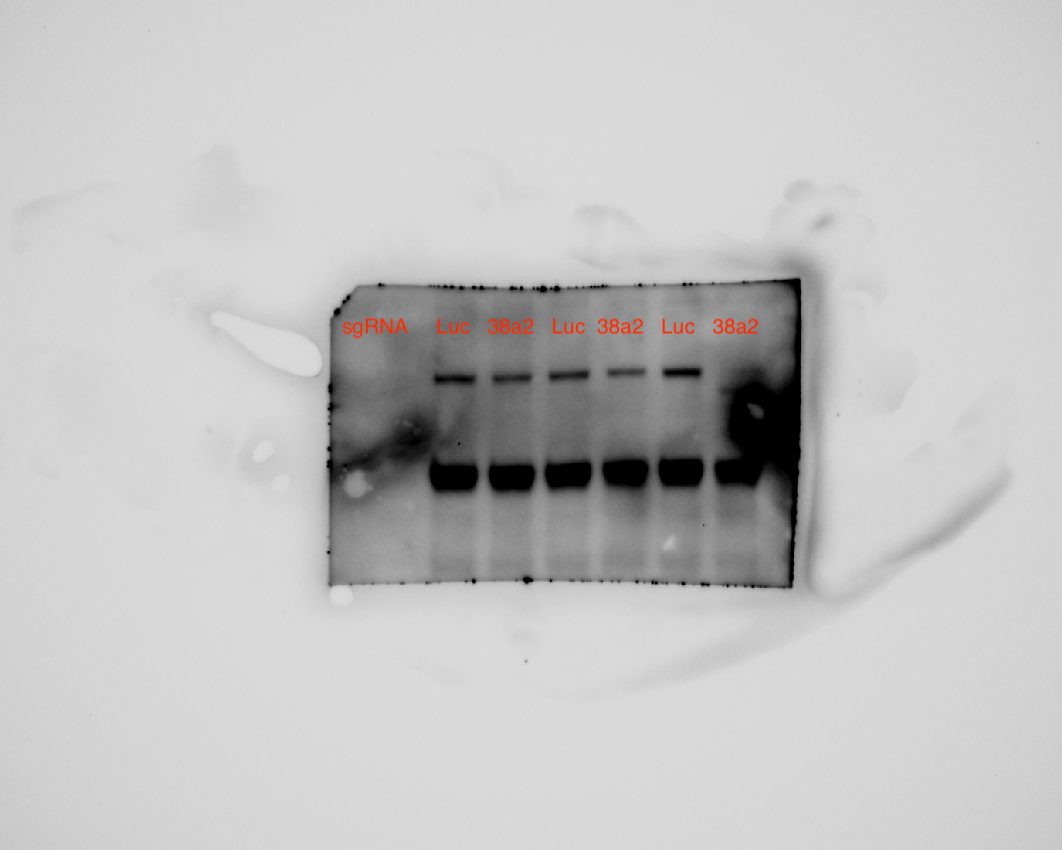

Supplement: Figure 4—source data 1. [file elife-76963-fig4-data1.zip › Figure 4-source data/Figure 4 C Western Blot Source Data/Figure 4C ATF4.tif]

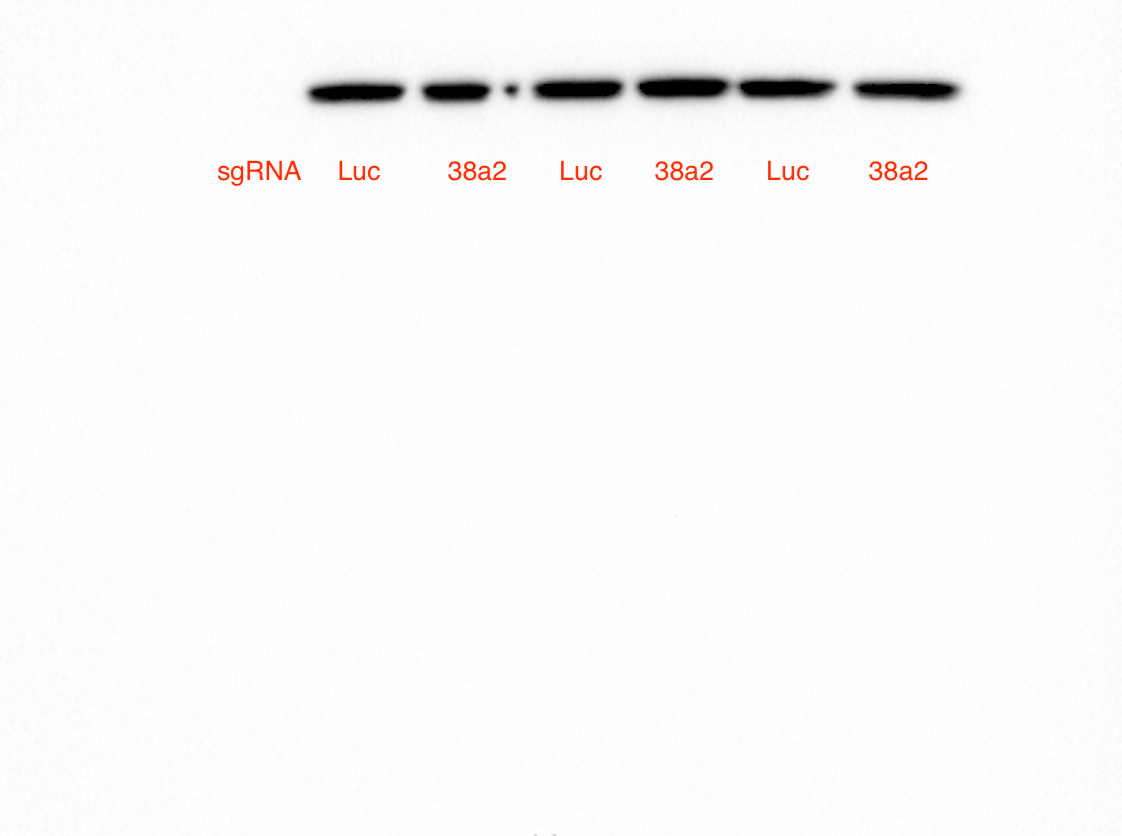

Supplement: Figure 4—figure supplement 1—source data 1. [file elife-76963-fig4-figsupp1-data1.zip › Figure 4 Supplement-source data/Figure 4 Supplement 1F Western Blot Source data/Figure 4 Supplement 1F pS6.tif]

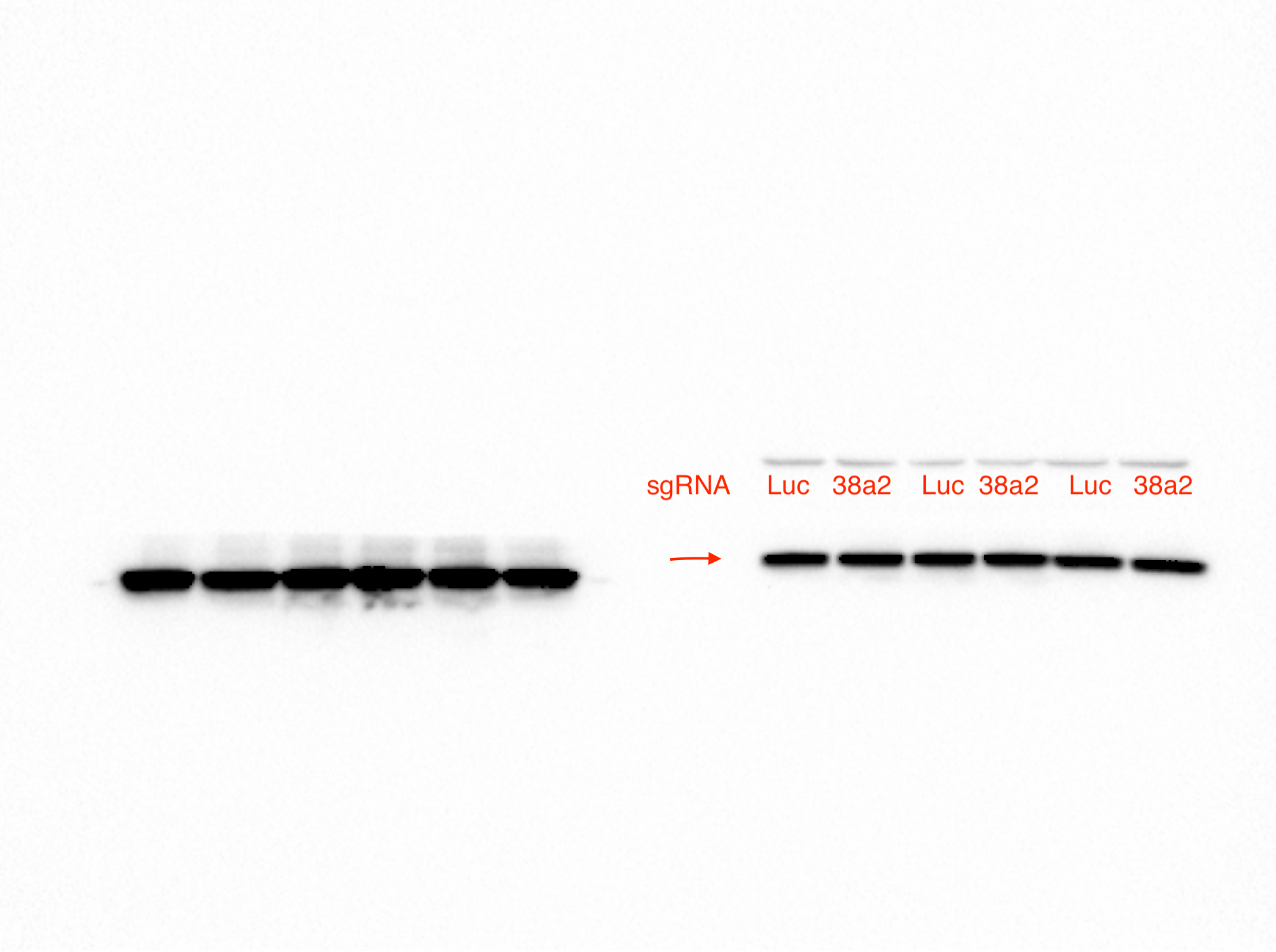

Supplement: Figure 4—figure supplement 1—source data 1. [file elife-76963-fig4-figsupp1-data1.zip › Figure 4 Supplement-source data/Figure 4 Supplement 1F Western Blot Source data/Figure 4 Supplement 1F S6.tif]

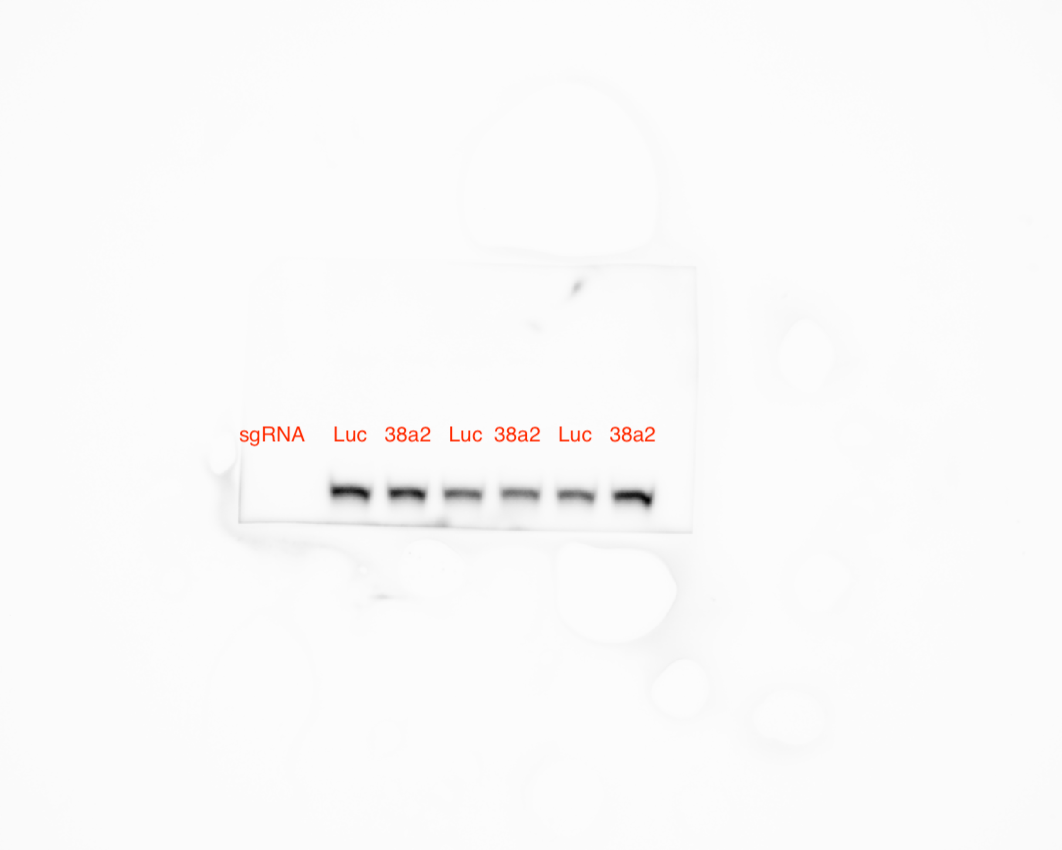

Supplement: Figure 4—figure supplement 1—source data 1. [file elife-76963-fig4-figsupp1-data1.zip › Figure 4 Supplement-source data/Figure 4 Supplement 1F Western Blot Source data/Figure 4 Supplement 1F PHGDH.tif]

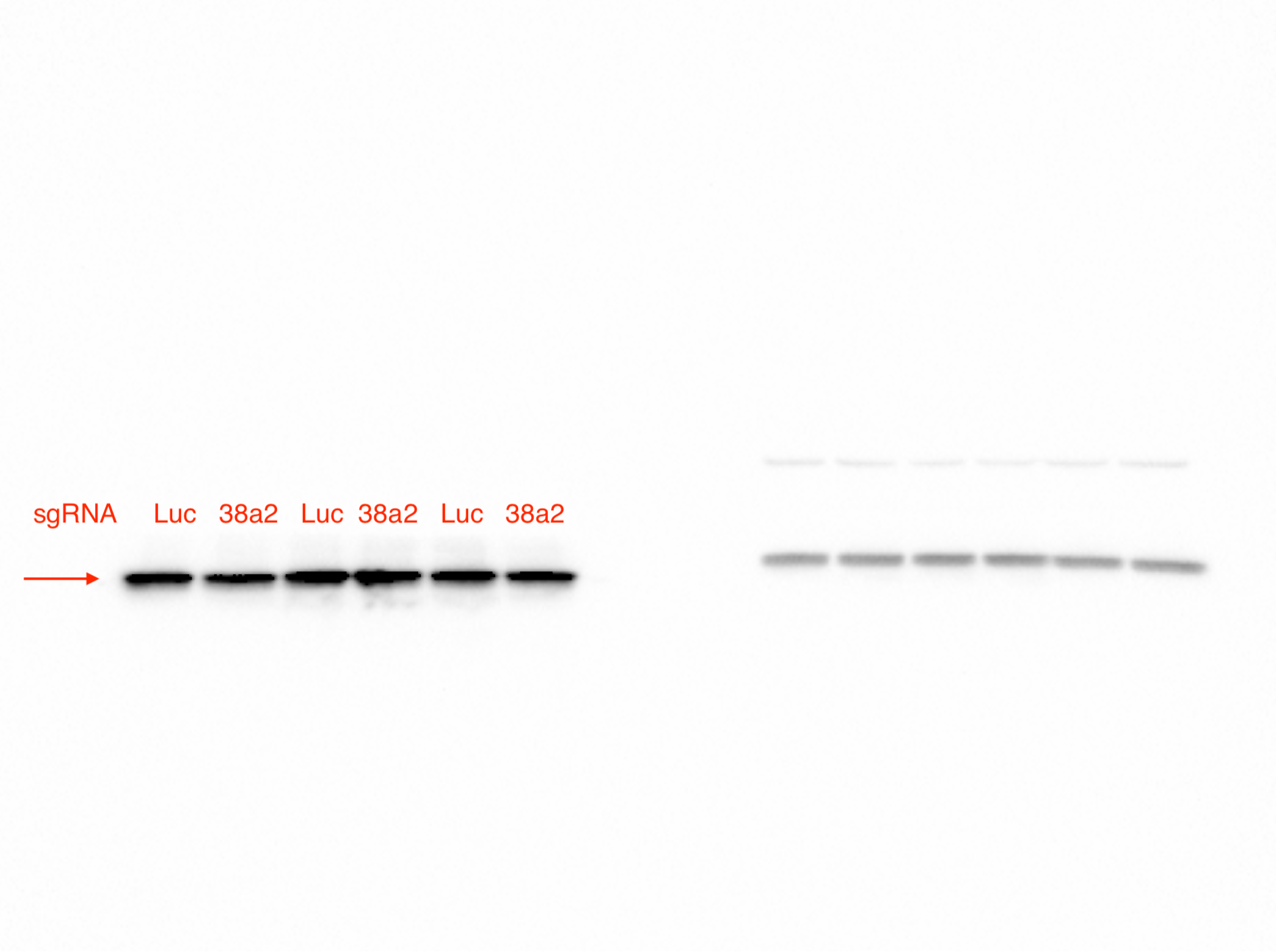

Supplement: Figure 4—figure supplement 1—source data 1. [file elife-76963-fig4-figsupp1-data1.zip › Figure 4 Supplement-source data/Figure 4 Supplement 1F Western Blot Source data/Figure 4 Supplement 1F EIF2A.tif]

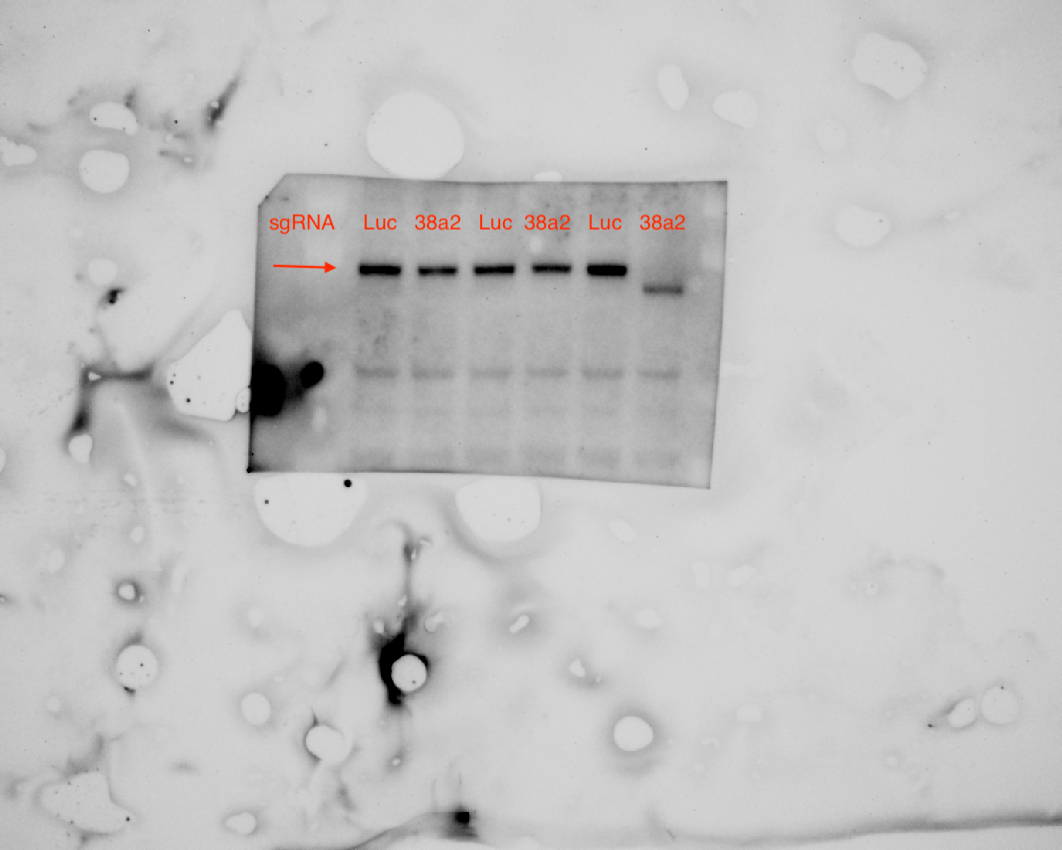

Supplement: Figure 4—figure supplement 1—source data 1. [file elife-76963-fig4-figsupp1-data1.zip › Figure 4 Supplement-source data/Figure 4 Supplement 1F Western Blot Source data/Figure 4 Supplement 1F PAX1.tif]

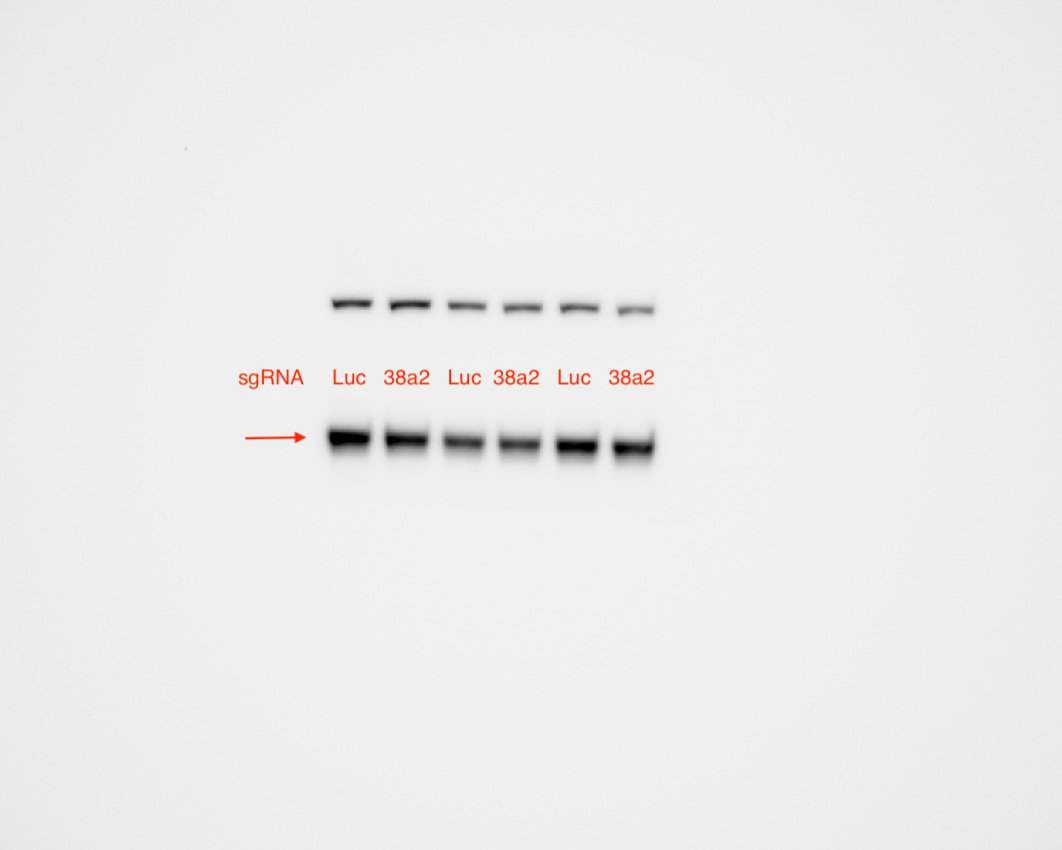

Supplement: Figure 4—figure supplement 1—source data 1. [file elife-76963-fig4-figsupp1-data1.zip › Figure 4 Supplement-source data/Figure 4 Supplement 1F Western Blot Source data/Figure 4 Supplement 1F AKT.tif]

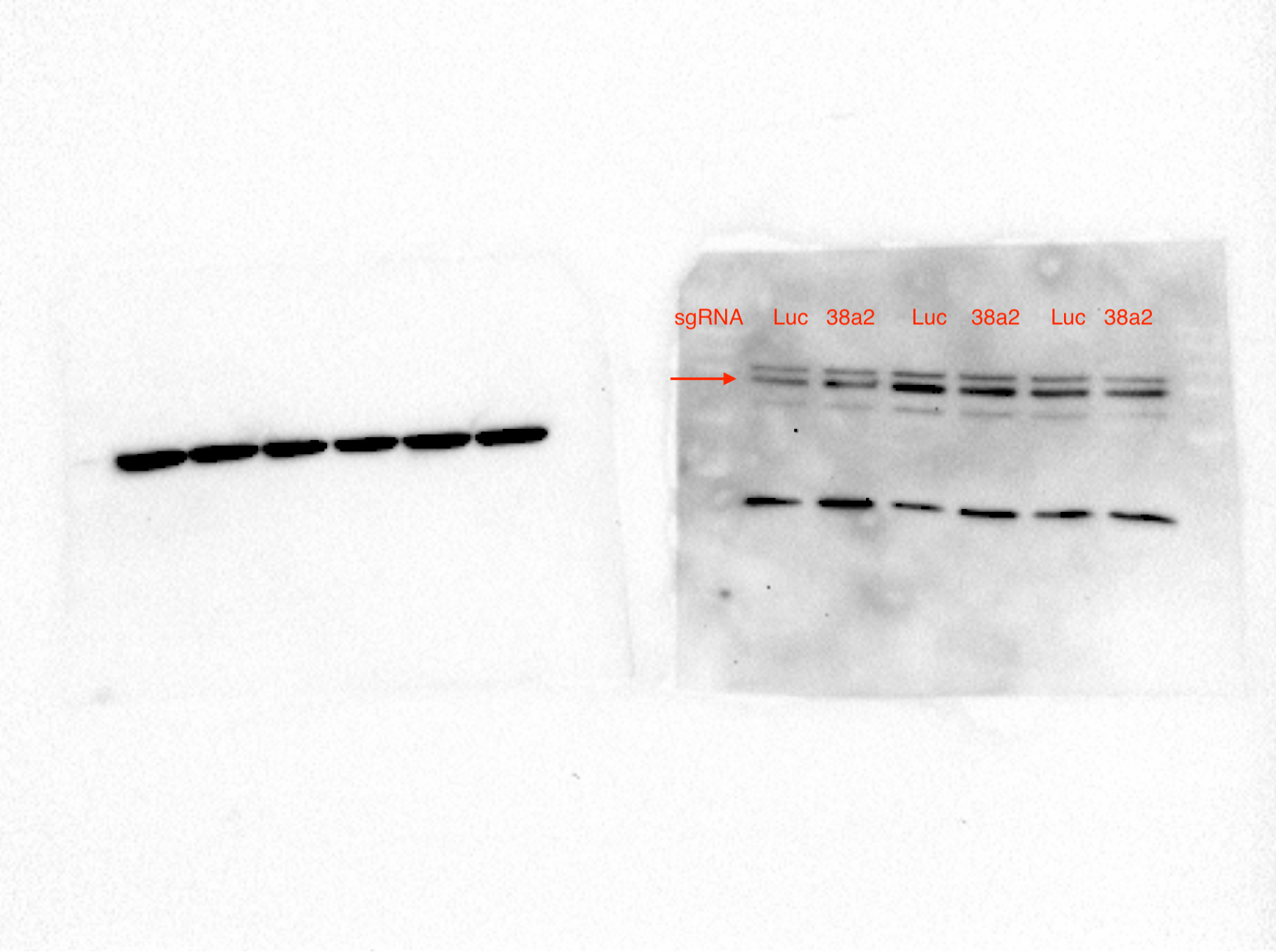

Supplement: Figure 4—figure supplement 1—source data 1. [file elife-76963-fig4-figsupp1-data1.zip › Figure 4 Supplement-source data/Figure 4 Supplement 1F Western Blot Source data/Figure 4 Supplement 1F ATF2.tif]

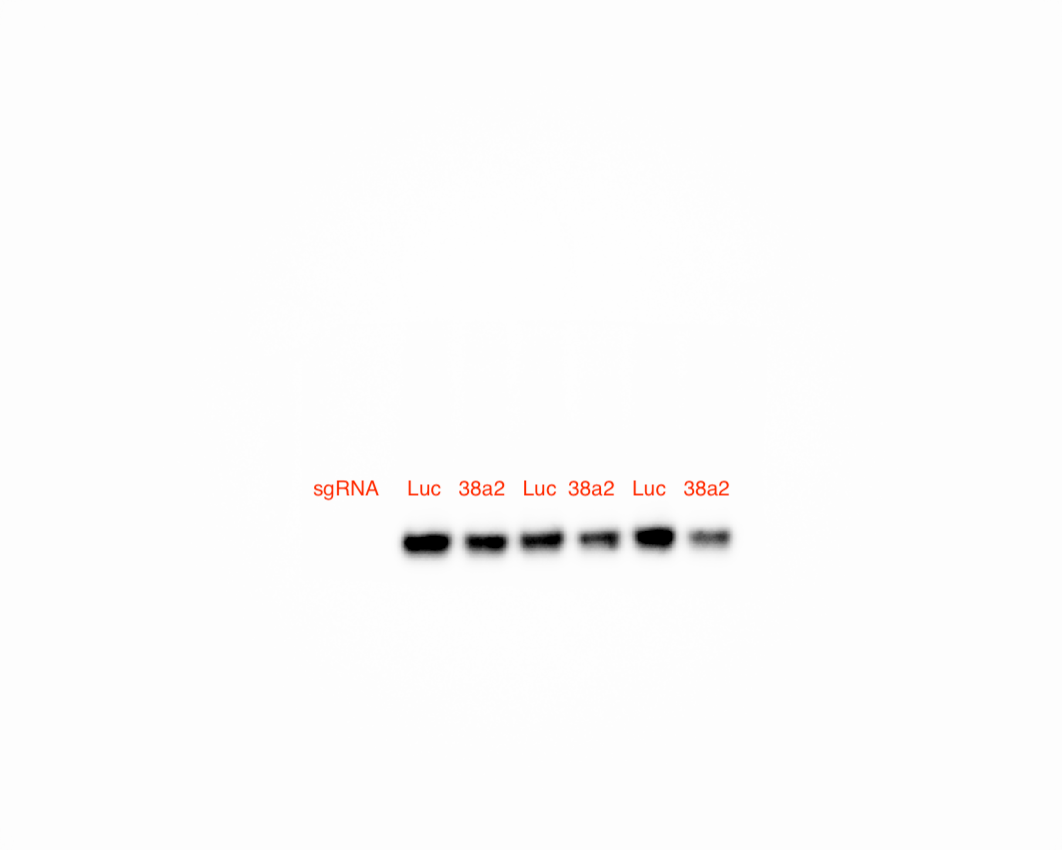

Supplement: Figure 4—figure supplement 1—source data 1. [file elife-76963-fig4-figsupp1-data1.zip › Figure 4 Supplement-source data/Figure 4 Supplement 1F Western Blot Source data/Figure 4 Supplement 1F EIF4EBP1.tif]

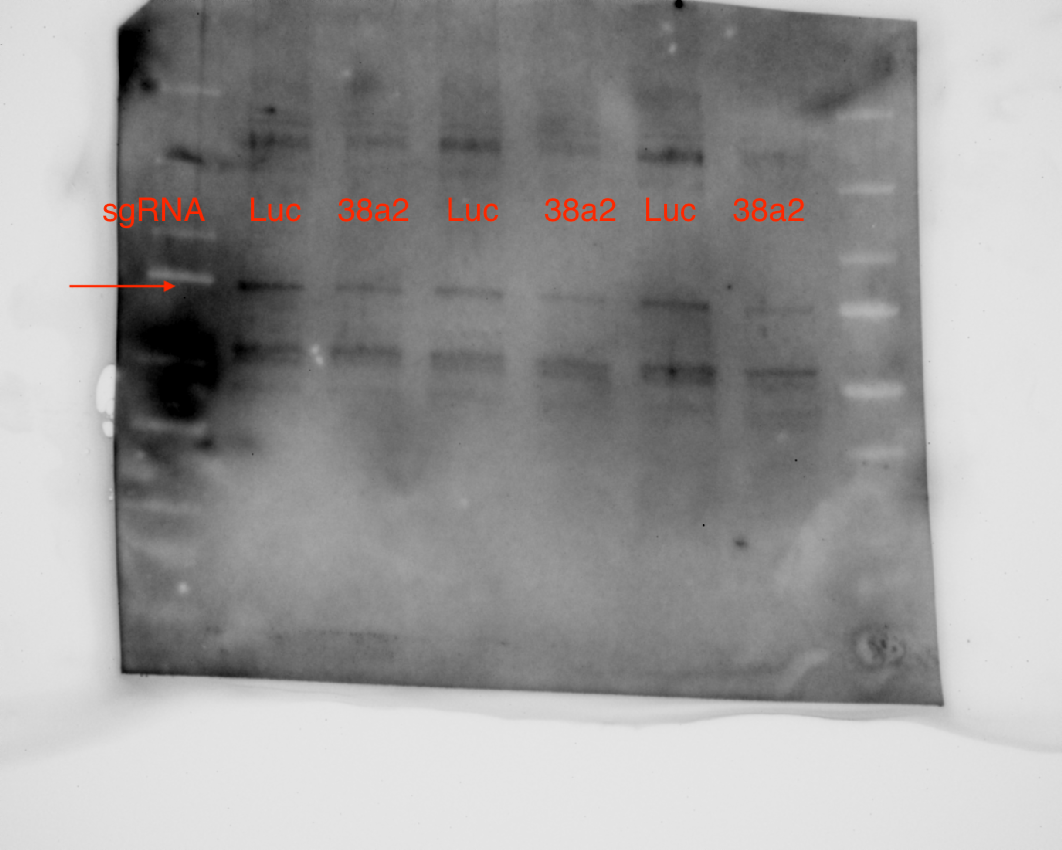

Supplement: Figure 4—figure supplement 1—source data 1. [file elife-76963-fig4-figsupp1-data1.zip › Figure 4 Supplement-source data/Figure 4 Supplement 1F Western Blot Source data/Figure 4 Supplement 1F OSX.tif]

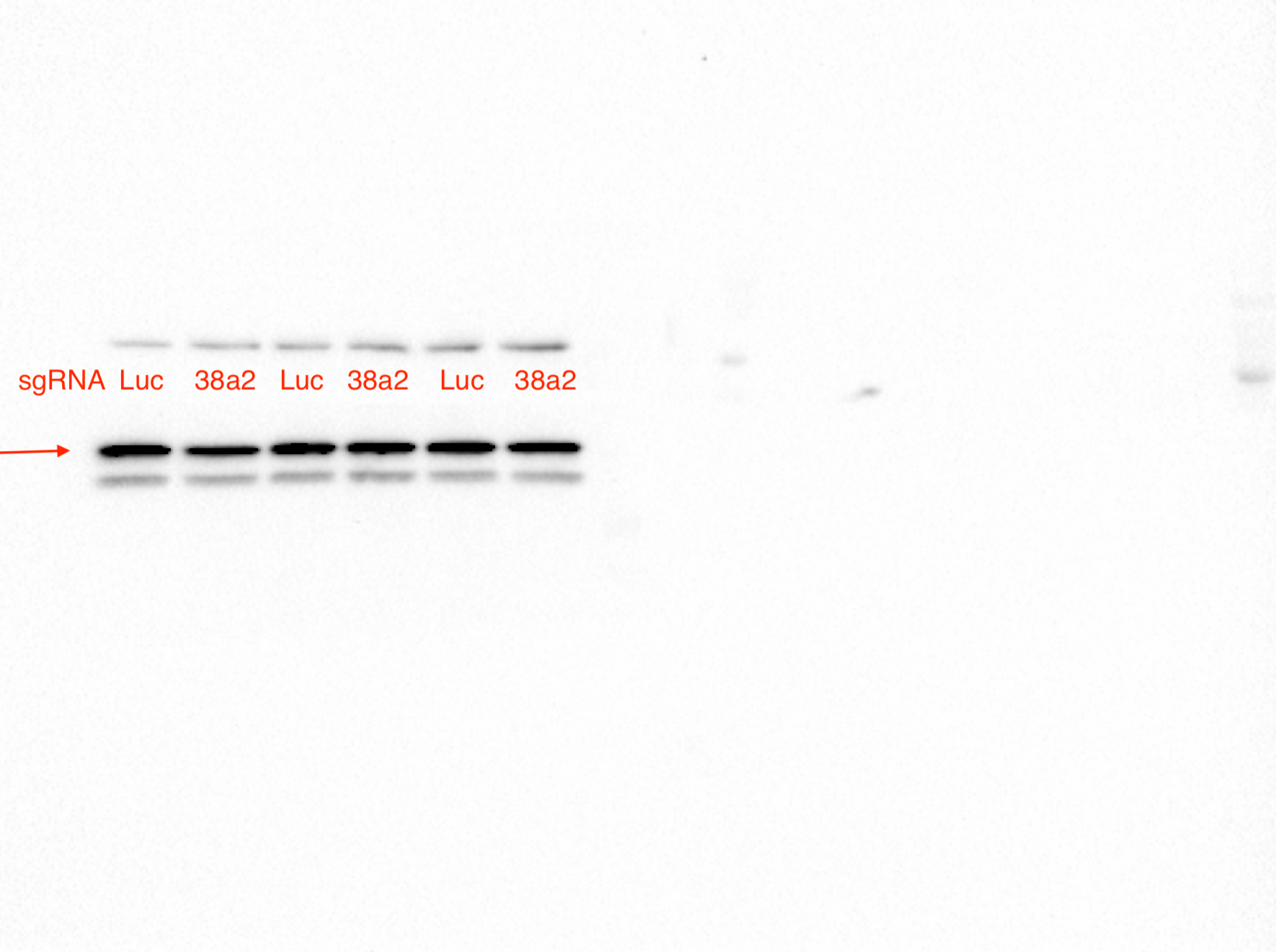

Supplement: Figure 4—figure supplement 1—source data 1. [file elife-76963-fig4-figsupp1-data1.zip › Figure 4 Supplement-source data/Figure 4 Supplement 1F Western Blot Source data/Figure 4 Supplement 1F pEif2a.tif]

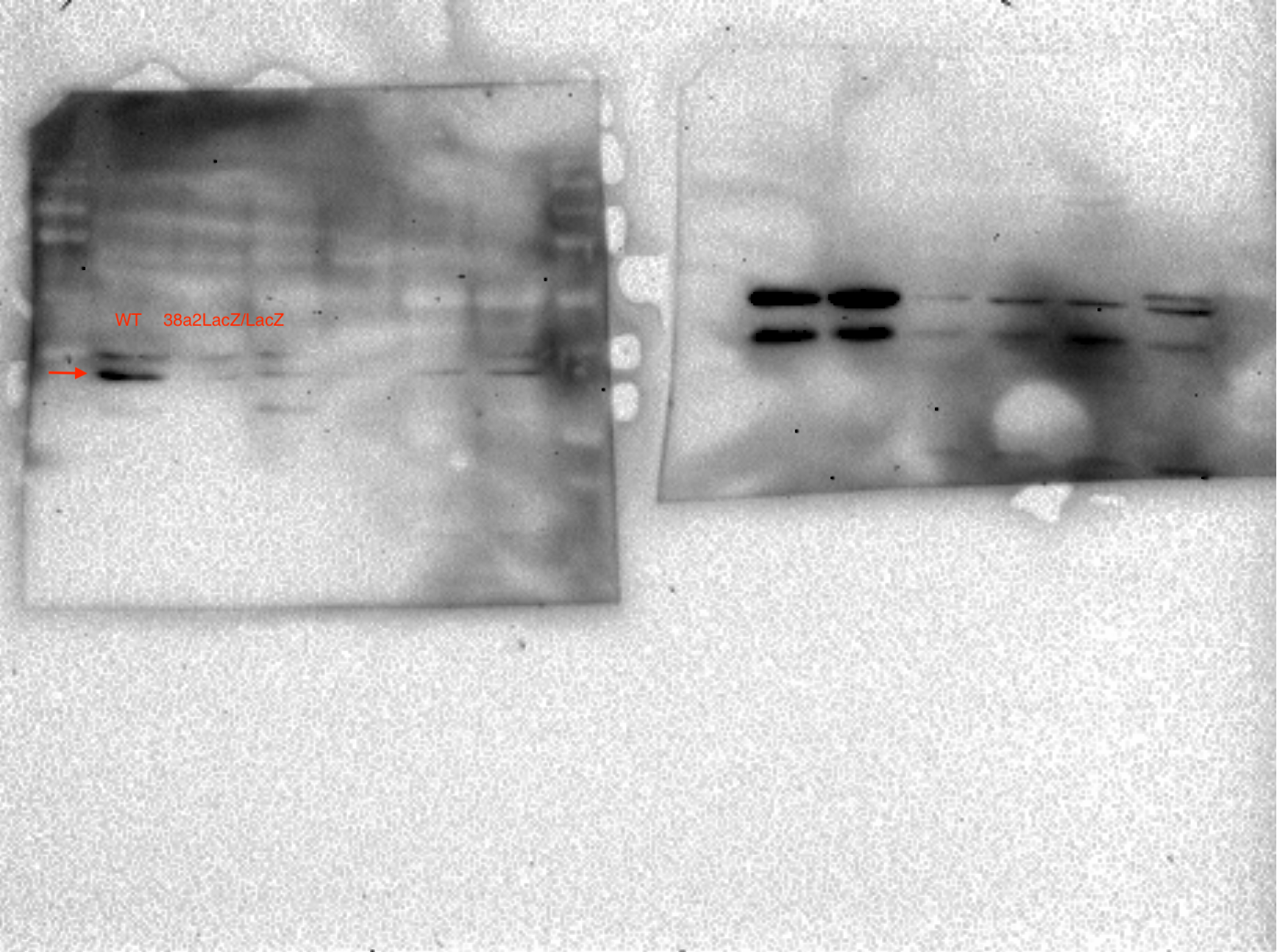

Supplement: Figure 5—figure supplement 2—source data 1. [file elife-76963-fig5-figsupp2-data1.zip › Figure 5 Supplement 2-source data/Figure 5 Supplement 2A Western Blot Source Data/Figure 5 Supplement 2A SNAT2.tif]

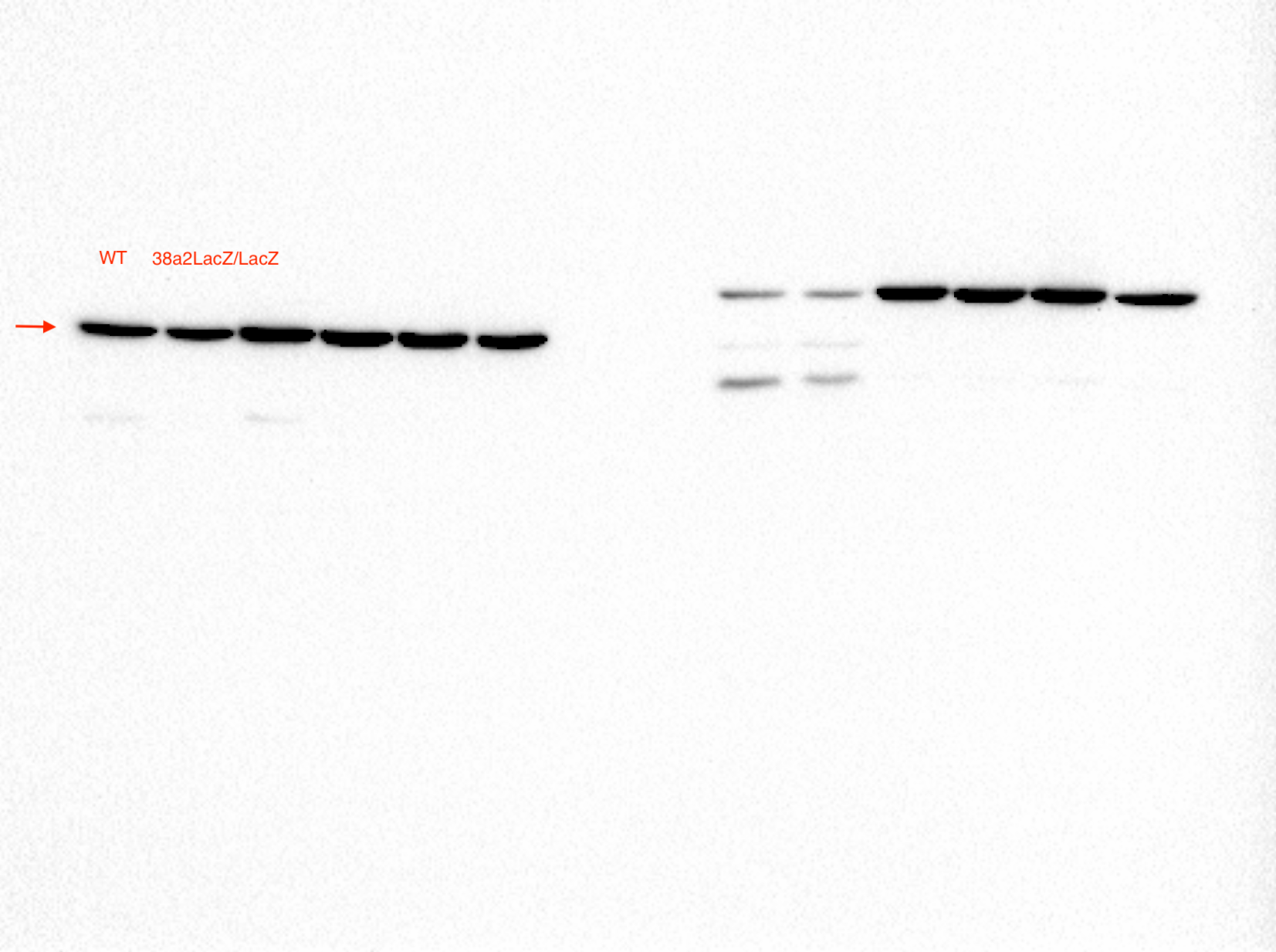

Supplement: Figure 5—figure supplement 2—source data 1. [file elife-76963-fig5-figsupp2-data1.zip › Figure 5 Supplement 2-source data/Figure 5 Supplement 2A Western Blot Source Data/Figure 5 Supplement 2A ACTB.tif]

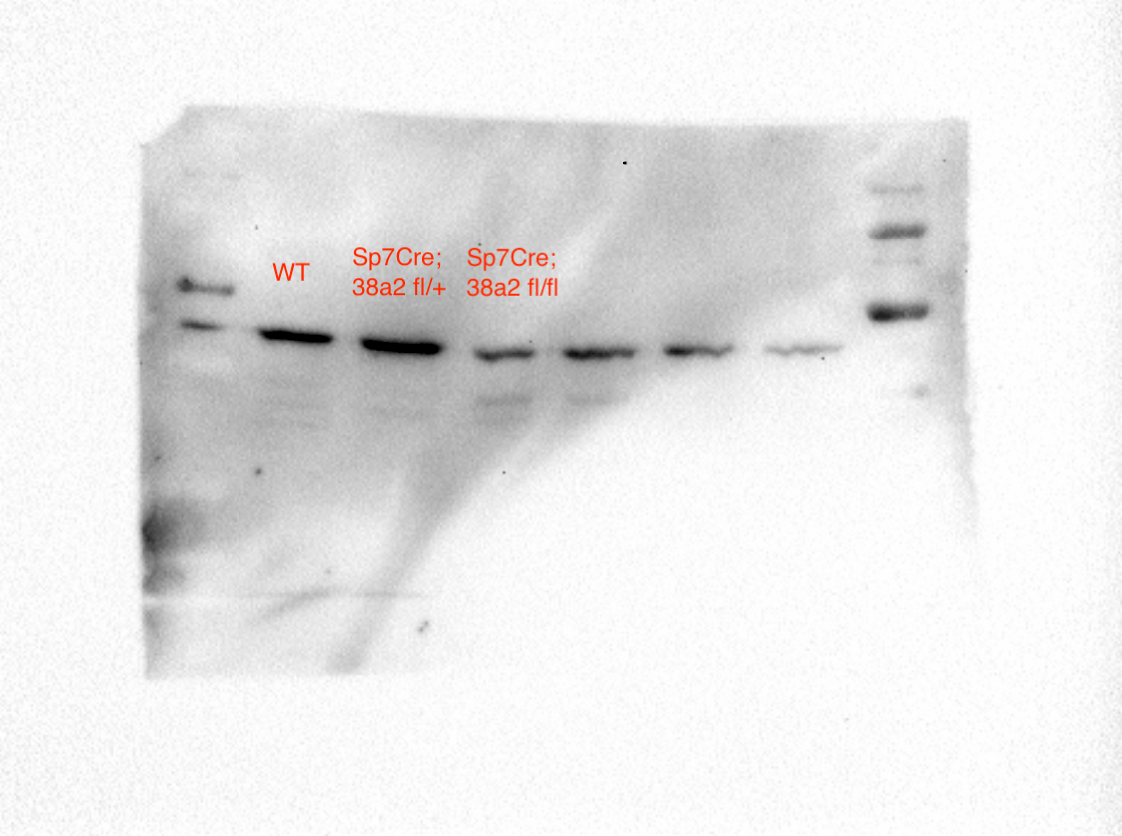

Supplement: Figure 5—figure supplement 3—source data 1. [file elife-76963-fig5-figsupp3-data1.zip › Figure 5 Supplement 3-source data/Figure 5 Supplement 3A Western Blot Source Data /Figure 5 Supplement 3A SNAT2.tif]

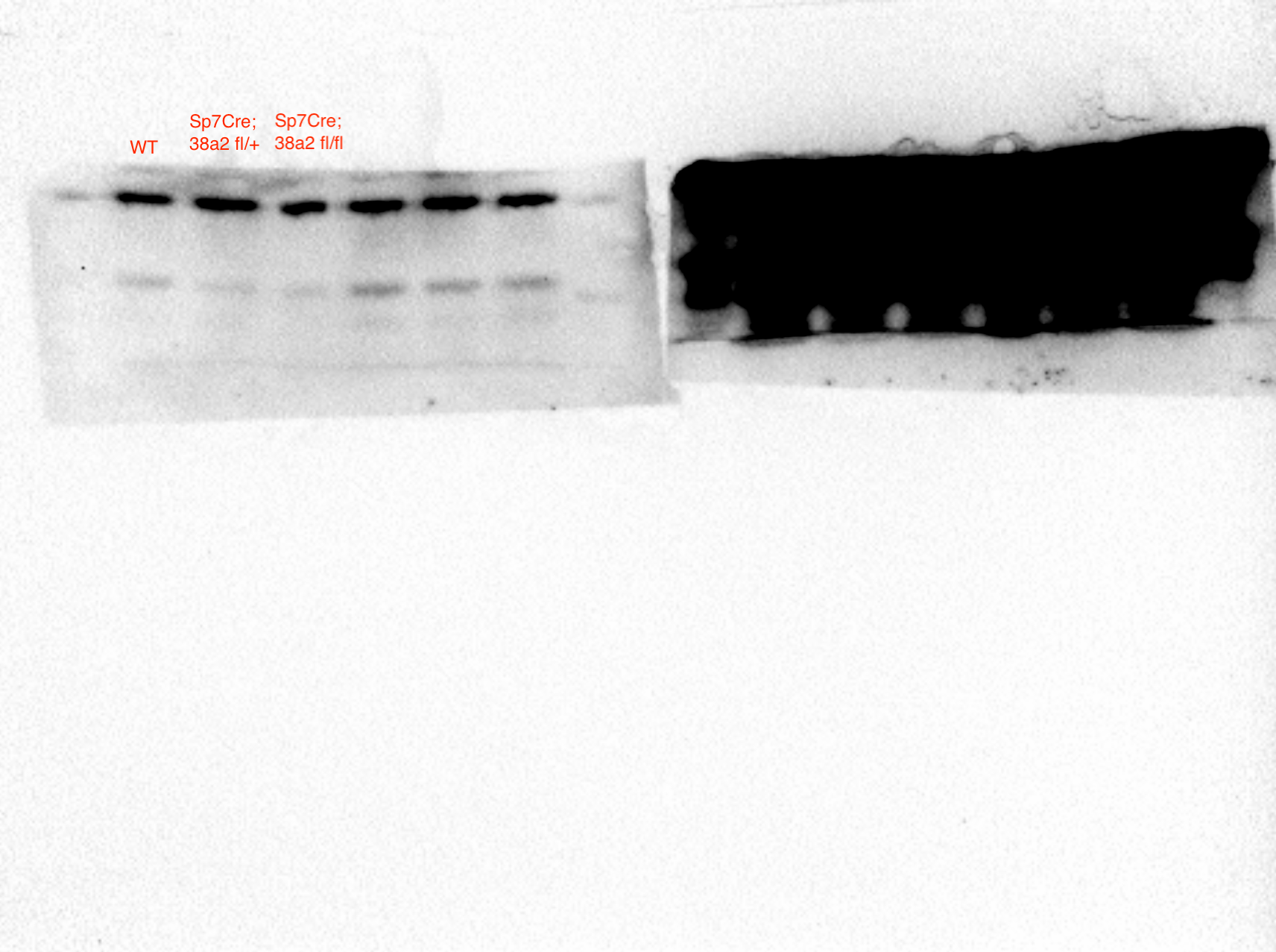

Supplement: Figure 5—figure supplement 3—source data 1. [file elife-76963-fig5-figsupp3-data1.zip › Figure 5 Supplement 3-source data/Figure 5 Supplement 3A Western Blot Source Data /Figure 5 Supplement 3A ACTB.tif]
