# Supplementary material for: SLC38A2 provides proline to fulfill unique synthetic demands arising during osteoblast differentiation and bone formation
Source: eLife. 2022 Mar 9;11:e76963. doi: 10.7554/eLife.76963 (PMC9007586; doi:10.7554/eLife.76963)
Supplement: Supplementary file 1. [file elife-76963-supp1.docx]

Supplementary File 1. sgRNA protospacer sequence

| SP498.mCherry.g17 | CAAGTAGTCGGGGATGTCGGNGG |
| --- | --- |
| SP498.mCherry.g19 | AGTAGTCGGGGATGTCGGCGNGG |
| SP499.Luc.g3 | CAATTCTTTATGCCGGTGTTNGG |
| SP399.Luc.g4 | GTGTTGGGCGCGTTATTTATNGG |
| MS347.Slc38a2.g1 | GTATCTGAACGGTGACTATCNGG |
| MS348.Slc38a2.g11 | GAGTTGAAGATGAAATAGCGNGG |
| MS348.Slc38a2.g13 | ATGCCAACGCCAACGCTGCCNGG |
| MS348.Slc38a2.g21 | AAGCAGCTTCCACGGGGCAANGG |
| MS348.Slc38a2.g4 | GTGGCCAACGAAACTGTGAANGG |
