# Supplementary material for: SLC38A2 provides proline to fulfill unique synthetic demands arising during osteoblast differentiation and bone formation
Source: eLife. 2022 Mar 9;11:e76963. doi: 10.7554/eLife.76963 (PMC9007586; doi:10.7554/eLife.76963)
Supplement: Supplementary file 2. [file elife-76963-supp2.docx]

Supplementary File 2. RT-PCR primer sequences for tRNA charging

| Amino acid | tRNA | Primer sequence |
| --- | --- | --- |
| Pro | AGG | GGCTCGTTGGTCTAGGGGTATG |
| Leu | CAG | GTCAGGATGGCCGAGCGGTCTA |
| Gln | TTG | GGTCCCATGGTGTAATGGTT |
| Glu | TTC | CCCACATGGTCTAGCGGTTA |
| Asn | GTT | GTCTCTGTGGCGCAATCGGT |
| Val | TAC | GGTTCCATAGTGTAGTGGTTAT |
| Reverse | | GCCTTGGCACCCGAGAATTCCA |
