# Supplementary material for: SLC38A2 provides proline to fulfill unique synthetic demands arising during osteoblast differentiation and bone formation
Source: eLife. 2022 Mar 9;11:e76963. doi: 10.7554/eLife.76963 (PMC9007586; doi:10.7554/eLife.76963)
Supplement: Supplementary file 3. [file elife-76963-supp3.docx]

Supplementary File 3. RT-PCR primer sequences

| Gene Symbol | Forward | Reverse |
| --- | --- | --- |
| β-actin | AGATGTGGATCAGCAAGCAG | GCGCAAGTTAGGTTTTGTCA |
| Akp2 | CCAACTCTTTTGTGCCAGAGA | GGCTACATTGGTGTTGAGCTTTT |
| Ibsp | CAGAGGAGGCAAGCGTCACT | GCTGTCTGGGTGCCAACACT |
| Bglap | CAGCGGCCCTGAGTCTGA | GCCGGAGTCTGTTCACTACCTTA |
| Sp7 | CCCTTCTCAAGCACCAATGG | AAGGGTGGGTAGTCATTTGCATA |
| Runx2 | CCAACCGAGTCATTTAAGGCT | GCTCACGTCGCTCATCTTG |
| Slc38a2 | GGCTATGTCAAGCTACCTCTTC | GTCACCGTTCAGATACCACAA |
| mTOR | AGAAGGGTCTCCAAGGACGACT | GCAGGACACAAAGGCAGCATTG |
| Smad1 | CTGAAGCCTCTGGAATGCTGTG | CAGAAGGCTGTGCTGAGGATTG |
| eEF2 | CAGAAGTACCGTTGTGAGCTGC | GTCAGAGGTTGGCACCATCTTG |
| Erk2 | TCAAGCCTTCCAACCTCCTGCT | AGCTCTGTACCAACGTGTGGCT |
| EIF4EBP1 | GGAGAGCTGCACAGCATTCAGG | GGAGGTATGTGCTGGTGTTCAC |
| Akt1 | GGACTACTTGCACTCCGAGAAG | CATAGTGGCACCGTCCTTGATC |
| Phgdh | CCTCCTTTGGTGTTCAGCAGCT | CGCACACCTTTCTTGCACTGAG |
| Tuba1 | GGCAGTGTTCGTAGACCTGGAA | CTCCTTGCCAATGGTGTAGTGG |
| Atf4 | GCATGCTCTGTTTCGAATGGA | CCAACGTGGTCAAGAGCTCAT |
| Atf2 | CTTCCTCTCCTCAACCAGTCCA | GAGTCCTAACCAATCCACTGCC |
| Pax1 | TCGCCAGCAGTGAATGGACTCG | ATACTCCGTGCTGGTTGGAAGC |
